# Phosphorylation of CRYAB induces a condensatopathy to worsen post–myocardial infarction left ventricular remodeling

**DOI:** 10.1172/JCI163730

**Published:** 2025-02-11

**Authors:** Moydul Islam, David R. Rawnsley, Xiucui Ma, Walter Navid, Chen Zhao, Xumin Guan, Layla Foroughi, John T. Murphy, Honora Navid, Carla J. Weinheimer, Attila Kovacs, Jessica Nigro, Aaradhya Diwan, Ryan P. Chang, Minu Kumari, Martin E. Young, Babak Razani, Kenneth B. Margulies, Mahmoud Abdellatif, Simon Sedej, Ali Javaheri, Douglas F. Covey, Kartik Mani, Abhinav Diwan

**Affiliations:** 1 Division of Cardiology and; 2 Center for Cardiovascular Research, Department of Medicine, Washington University School of Medicine, St. Louis, Missouri, USA.; 3 Department of Chemistry, Washington University in St. Louis, St. Louis, Missouri, USA.; 4 Division of Cardiology and Department of Medicine, University of Alabama, Birmingham, Alabama, USA.; 5 John Cochran Veterans Affairs Medical Center, St. Louis, Missouri, USA.; 6 Department of Medicine, University of Pittsburgh, Pittsburgh, Pennsylvania, USA.; 7 Department of Medicine, University of Pennsylvania, Philadelphia, Pennsylvania, USA.; 8 Division of Cardiology, Medical University of Graz, Graz, Austria.; 9 BioTechMed-Graz, Graz, Austria.; 10 Institute of Physiology, University of Maribor, Maribor, Slovenia.; 11 Department of Developmental Biology and; 12 Department of Anesthesiology, Psychiatry, and Taylor Family Institute for Innovative Psychiatric Research, Washington University in St. Louis, St. Louis, Missouri, USA.; 13 Cardiovascular Service Line, HCA Midwest Health, Overland Park, Kansas, USA.; 14 Departments of Cell Biology and Physiology, Obstetrics and Gynecology, and Neurology, Washington University in St. Louis, St. Louis, Missouri, USA.

**Keywords:** Cardiology, Cell biology, Cardiovascular disease, Chaperones

## Abstract

Protein aggregates are emerging therapeutic targets in rare monogenic causes of cardiomyopathy and amyloid heart disease, but their role in more prevalent heart-failure syndromes remains mechanistically unexamined. We observed mislocalization of desmin and sarcomeric proteins to aggregates in human myocardium with ischemic cardiomyopathy and in mouse hearts with post–myocardial infarction ventricular remodeling, mimicking findings of autosomal-dominant cardiomyopathy induced by the R120G mutation in the cognate chaperone protein CRYAB. In both syndromes, we demonstrate increased partitioning of CRYAB phosphorylated on serine 59 to NP40-insoluble aggregate-rich biochemical fraction. While CRYAB undergoes phase separation to form condensates, the phosphomimetic mutation of serine 59 to aspartate (S59D) in CRYAB mimics R120G-CRYAB mutants with reduced condensate fluidity, formation of protein aggregates, and increased cell death. Conversely, changing serine to alanine (phosphorylation-deficient mutation) at position 59 (S59A) restored condensate fluidity and reduced both R120G-CRYAB aggregates and cell death. In mice, S59D CRYAB knockin was sufficient to induce desmin mislocalization and myocardial protein aggregates, while S59A CRYAB knockin rescued left ventricular systolic dysfunction after myocardial infarction and preserved desmin localization with reduced myocardial protein aggregates. 25-Hydroxycholesterol attenuated CRYAB serine 59 phosphorylation and rescued post–myocardial infarction adverse remodeling. Thus, targeting CRYAB phosphorylation-induced condensatopathy is an attractive strategy to counter ischemic cardiomyopathy.

## Abstract 2

CRYAB, a cardiac-myocyte enriched chaperone protein, forms condensates in physiology. CRYAB phosphorylation post-myocardial infarction triggers abnormal condensates to provoke protein aggregates and cause heart failure.

## Introduction

Cardiomyopathy is characterized by altered structure and function of the cardiac contractile apparatus, whereby targeted approaches to restore sarcomere structure and function hold therapeutic promise. While genetic mutations in contractile proteins reveal mechanisms that have spurred development of myosin modulators to treat hypertrophic cardiomyopathy (1), mechanistic studies targeting the cardiac contractile apparatus in more common causes of cardiomyopathy are lacking. As a case in point, myocardial infarction (MI) and ischemia are implicated in the pathogenesis of nearly two-thirds of all cases of cardiomyopathy and heart failure; and abnormal sarcomere function (2) with transcriptional (3) and posttranslational modifications of sarcomeric proteins (4, 5) has been described in ischemic cardiomyopathy (ICM). Similarly, studies have demonstrated loss of sarcomeres with decompensation of pressure overload hypertrophy to develop cardiac dysfunction (6), a common pathophysiologic mechanism implicated in causing heart failure. Given that state-of-the art strategies to treat cardiomyopathy, including myocardial reperfusion, neurohormonal antagonism, and resynchronization, are only partially effective in improving outcomes (7), there is an urgent unmet need to treat the pathology affecting the cardiac myocyte contractile apparatus in these highly prevalent causes of cardiomyopathy.

Cardiac myocytes rely on intricately coordinated protein quality control mechanisms to maintain structure and function and drive uninterrupted sarcomere shortening and relaxation that underlies cardiac function (8). These homeostatic mechanisms respond rapidly to stress, regulating various steps over the life cycle of individual proteins. Chaperone proteins play a central role in maintaining cardiac myocyte protein quality, and mutations in CRYAB (HSPB5), HSP27 (HSPB7), HSPB6, HSPB8, and cochaperones such as BAG3 are implicated in the pathogenesis of human cardiomyopathies by affecting stability, localization, or turnover of sarcomeric proteins (9). Mechanisms that drive cardiomyopathy in these rare genetic disorders may offer clues to understanding the molecular basis for sarcomeric abnormalities in more common causes of heart failure and inform novel approaches for prevention and treatment of associated morbidity and mortality.

Intriguingly, many disease-associated mutations in cardiac chaperone proteins induce intracellular protein-aggregate pathology in cardiac myocytes (10–12). Cardiac myocyte protein-aggregate pathology is also observed with human idiopathic dilated cardiomyopathy, ICM, and hypertrophic cardiomyopathy (13–16). Desmin, a sarcomere-associated protein, plays a critical role in maintenance of sarcomere structure and subcellular registration; and mutations in desmin or its chaperone CRYAB result in functional deficiency of desmin or its mislocalization to aggregates, resulting in a group of disorders termed “desminopathies” (17). In some studies, reduced phosphorylation of desmin and its cleavage have also been associated with development of pressure-overload–induced cardiomyopathy (16). We and others have demonstrated that the mutant R120G protein binds with increased affinity with desmin to sequester it in aggregates (18, 19), and stimulation of the autophagy-lysosome pathway removes CRYAB-R120G mutant proteins and its aggregates and restores normal desmin localization (18, 20). Notably, this phenomenon of aggregate-prone proteins hijacking normal cardiac myocyte proteins is also observed with expression of P209L mutation of human BAG3, which induces restrictive cardiomyopathy in humans (21). Transgenic expression of P209L BAG3 in the mouse heart induced protein aggregates, with sequestration of endogenous WT BAG3 as well as sarcomere-associated proteins such as desmin, α-actinin, and myopodin (SYNPO-2) in aggregates, provoking sarcomere disruption and cardiomyopathy (22). Therefore, examining the mechanisms by which proteins become aggregate prone holds therapeutic promise to prevent proteotoxic cardiac dysfunction.

To understand the mechanisms by which chaperone-protein interactions are pathologically altered from dynamic protein assemblies in physiology to persistent protein aggregates in pathology (8), we focused on the phenomenon of phase separation of biomolecules, to form membrane-less compartments termed “condensates” (23). The biophysical principles of phase separation rely on multivalent interactions, realized by oligomerization of intrinsically disordered regions (IDRs) within proteins that do not display a fixed tertiary structure and combine with similar domains in proteins or interact with other biomolecules (24). Evidence is accumulating that mutations or protein modifications cause biomolecular condensates to transition from a liquid state to a gel-like state to nucleate conversion to protein aggregates that are observed in a variety of disease states (25, 26). We discovered that CRYAB undergoes phase separation and stress-induced phosphorylation at serine 59, which is also increased in the aggregate-prone human-disease–causing R120G mutant. This alters liquid-like properties of CRYAB condensates toward gel-like behavior with reduced fluidity and aggregate formation, conferring cellular toxicity. Treatment with 25-hydroxycholesterol (25-HC), a compound that ameliorates CRYAB-R120G aggregates in the lens (27), limited stress-induced phosphorylation of CRYAB and the resultant improvement in condensate properties was associated with attenuation of post-MI ventricular remodeling. Taken together, our data demonstrate a mechanistic role of altered condensate behavior of CRYAB, i.e., a condensatopathy (28), in the pathogenesis of ICM and support the development of pharmacologic approaches to reduce the abundance of this aggregate-prone toxic protein to treat this common cause of heart failure.

## Results

### Desmin mislocalizes to protein aggregates in human ICM.

**Figure 1:**
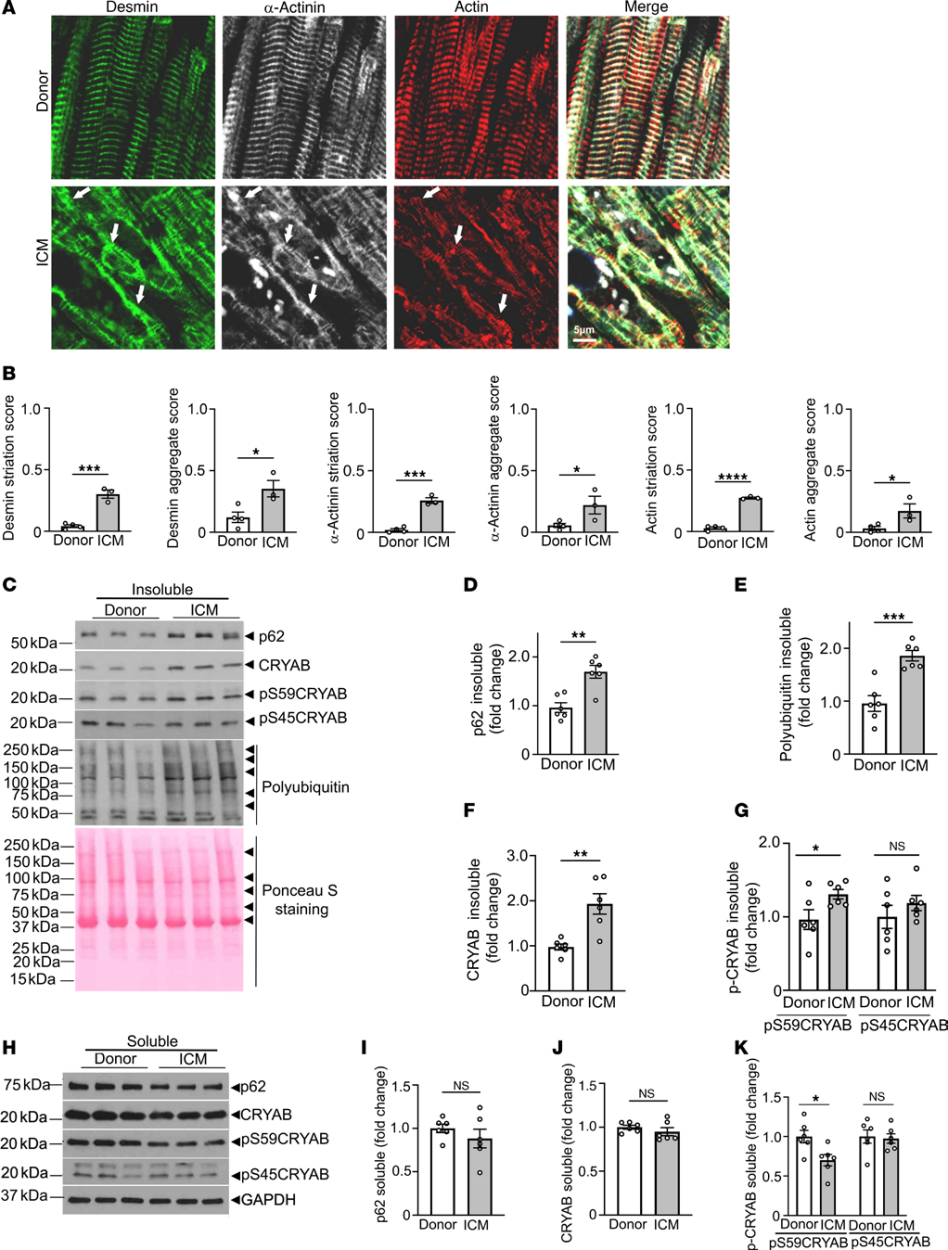
Desmin, α-actinin, and actin and the serine 59 phosphorylated form of their chaperone protein, CRYAB, localize to protein aggregates in human ICM. (**A**) Representative immunohistochemical images from left ventricular myocardium of individuals evaluated as controls (donor) or patients with end-stage ICM stained for desmin, α-actinin, and actin. Arrows point to mislocalization of these proteins from their physiologic location on Z-discs and intercalated discs (desmin), Z-disc (α-actinin), and sarcomere (actin) in donor myocardium to protein aggregates in ICM myocardium. (**B**) Quantitation of striation score and aggregate score for desmin, α-actinin, and actin in ICM and donor hearts. *n* = 3-4 hearts/group. For striation scoring, normal localization of proteins got scored as 0, and abnormal striation or mislocalization of proteins was scored as 1. For scoring aggregates, absence of aggregates was scored as 0 and presence of aggregates was scored as 2. (**C**–**G**) Immunoblot (**C**) and quantitation (fold change as compared with donor mean) depicting total p62 (**D**), polyUb proteins (**E**), CRYAB (**F**), and pS59-CRYAB and pS45-CRYAB (**G**) in NP40-detergent-insoluble fractions from human hearts from patients with ICM and donors. Ponceau S staining is shown as loading control. (**H**–**K**) Immunoblot (**H**) and quantitation for p62 (**I**), CRYAB (**J**), and pS59-CRYAB and pS45-CRYAB (**K**) abundance in NP-40 detergent soluble biochemical fractions from human hearts as in **C**–**G**. GAPDH was used as loading control. *n* = 6 samples/group for **C**–**K**. *P < 0.05; **P < 0.01; ***P < 0.001 versus donor as control by *t* test.

Mislocalization of desmin to protein aggregates is observed with heritable mutations in *DES*, the gene coding for desmin (17, 29), and in *CRYAB* (10), the gene coding for its chaperone protein, αB-crystallin (CRYAB). In these disorders, loss of physiologic desmin localization and consequently its function induces cardiomyopathy that mimics the pathology observed with experimental mouse ablation of the *DES* gene in mice (30), whereby these disorders are termed desminopathies (17). To examine desmin localization in ICM, we performed immunohistochemistry and biochemical subcellular fractionation on human heart tissue obtained from humans with ICM and from donor hearts without known cardiac pathology that were not used for transplantation (Supplemental Table 1; supplemental material available online with this article; https://doi.org/10.1172/JCI163730DS1). As shown in Figure 1A, desmin was observed along the Z-discs and intercalated discs in donor hearts, consistent with its physiologic localization. In contrast, desmin was mislocalized (with reduced localization in striations assessed as worse striation score, see legend) to protein aggregates in ICM hearts (Figure 1, A and B) along with polyubiquitinated (polyUb) proteins (Supplemental Figure 1A). This was accompanied by immunodetectable preamyloid oligomers in ICM hearts as detected by A11 antibody (15) staining (Supplemental Figure 1B). Importantly, we also detected mislocalization of actin, another CRYAB client protein (31), as well as of α-actinin (a Z-disc protein that is observed to interact with CRYAB; ref. 32), with increased localization of these proteins to aggregates in ICM hearts (Figure 1, A and B). This was accompanied by increased abundance of p62 (an adaptor protein that binds to and sequesters polyUb proteins in aggregates. refs. 33, 34) (Figure 1, C and D) and increased polyUb proteins (Figure 1, C and E) in the aggregate-rich NP40 detergent-insoluble fraction, suggesting sequestration of cardiac myocyte proteins in aggregates in ICM hearts.

To examine potential mechanisms for mislocalization of sarcomeric (actin, α-actinin) and sarcomere-associated proteins (desmin), we evaluated the localization of CRYAB, which plays an important role in chaperoning these proteins (19). We observed increased partitioning of CRYAB to the detergent-insoluble aggregate-rich fraction (Figure 1, C and F). Interestingly, prior studies indicate that CRYAB undergoes posttranslational modifications, specifically phosphorylation at serine resides (at position 19, 45, and 59), under stress (35). Of these, serine 45 and 59 are phosphorylated by stress-induced p38 MAPK (35) as well as by protein kinase N, which are activated in cardiac ischemia-reperfusion (IR) injury (36, 37). Also, studies in cell-culture models show that aggregate-prone R120G mutant of CRYAB is hyperphosphorylated at these 3 serine residues, which regulates its tendency to aggregate (38). Our findings demonstrate increased abundance of phosphorylated CRYAB at residue serine 59 (pS59-CRYAB), but not at serine 45 in the detergent-insoluble fraction (Figure 1, C and G) with a concomitant decline in its abundance in the soluble fraction (Figure 1, H–K), which parallels the partitioning of p62 and polyUb proteins to the aggregate-rich detergent-insoluble fraction.

To examine whether partitioning of pS59-CRYAB is also observed in mouse myocardium under stress, we performed closed-chest cardiac IR injury in young adult WT C57BL/6J mice and evaluated them 4 weeks later. As shown in Supplemental Figure 2, A and B, IR injury induced a marked increase in left ventricular end-diastolic volume (EDV) with decline in left ventricular ejection fraction (EF) at 4 weeks after MI, as compared with sham-operated mice, consistent with development of ICM. Similar to our observations in human ICM (Figure 1), this was accompanied by a significant increase in abundance of pS59-CRYAB with increased p62 in the aggregate-rich NP40-insoluble fraction (Supplemental Figure 2, C–I) and mislocalization of desmin, α-actinin, and actin in the post-IR myocardium (Supplemental Figure 2, J and K). Taken together, these findings demonstrate that CRYAB is increasingly phosphorylated at serine 59 in the setting of ICM in mice and in humans and partitions into the aggregate-rich NP40-insoluble biochemical protein fraction. This associated mislocalization of desmin (and other CRYAB clients) to protein aggregates suggests the hypothesis that phosphorylation at serine 59 may render CRYAB aggregate prone and sequester its client proteins within protein aggregates in ICM. Indeed, the aggregate-prone CRYAB-R120G mutant protein is phosphorylated at serine 59 in the mouse myocardium in transgenic R120G mice (Supplemental Figure 3), confirming prior observations in cell culture (38), in a mouse model for proteotoxic cardiomyopathy that recapitulates human pathology with mislocalization of desmin to protein aggregates (18) to induce a desminopathy. Taken together with our previous observation of increased affinity for R120G mutant protein to bind to desmin as compared with native CRYAB (18), these findings suggest the hypothesis that increased pS59-CRYAB binds to its client proteins with increased affinity to sequester them in aggregates in ICM.

### Phosphorylation at serine 59 is necessary and sufficient to make CRYAB aggregate prone.

**Figure 2:**
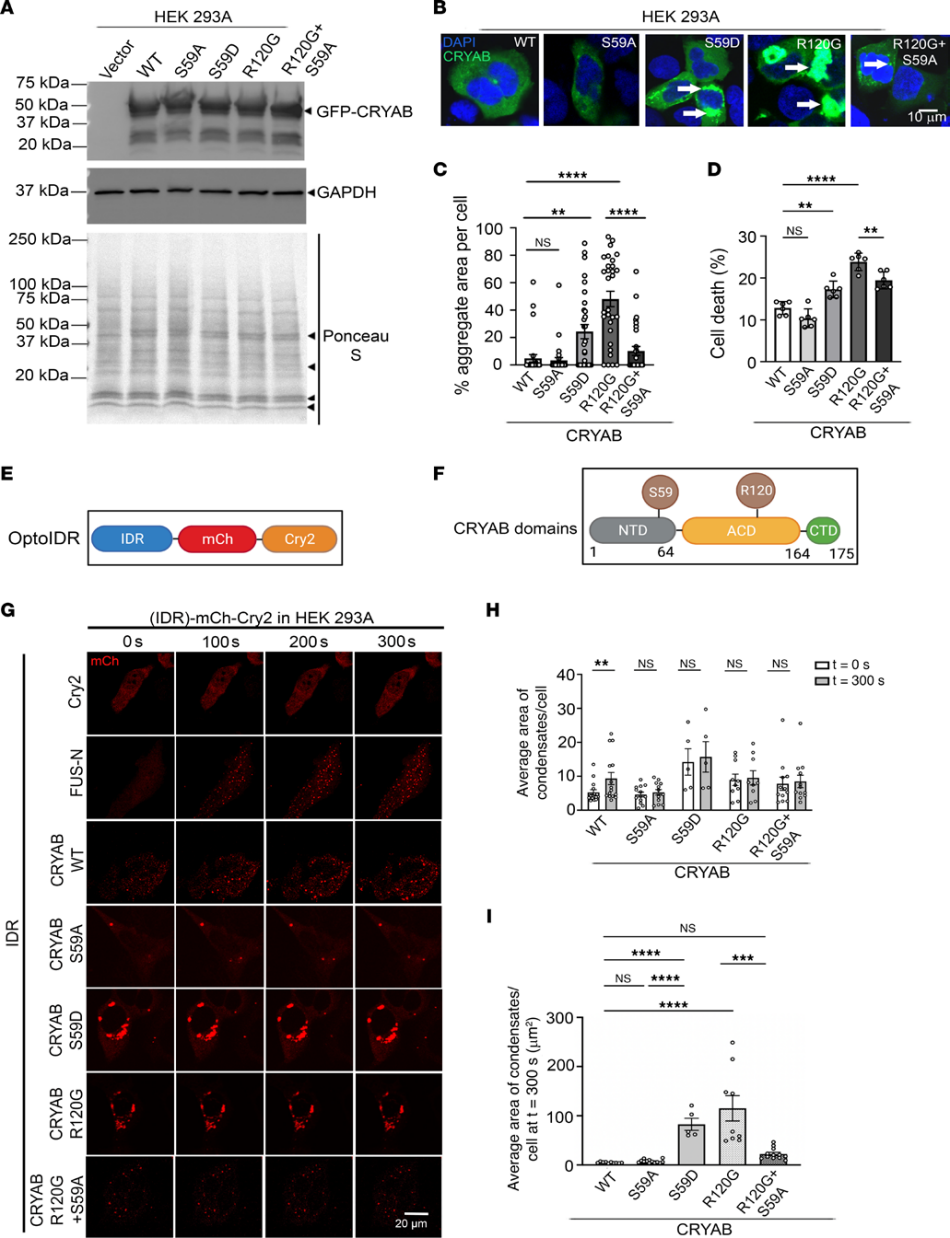
Phosphorylation of CRYAB at S59 makes it aggregate prone and toxic. (**A**) Immunoblot (**A**) demonstrating expression of GFP-fusion proteins in HEK293A cells transfected with GFP-tagged WT CRYAB, its phospho-mimetic mutant (S59D), phosphorylation-deficient mutant (S59A), R120G mutant, or the R120G and S59A double-mutant proteins. (**B** and **C**) Representative immunofluorescence images (**B**) for detection of protein aggregates with quantitation (**C**) of aggregate area per cell. **P < 0.01; ****P < 0.0001 by Tukey’s post hoc test after 1-way ANOVA. Nuclei are blue (DAPI). (**D**) Cell death in cells treated in *A*. **P < 0.01; ****P < 0.0001 by Tukey’s post hoc test after 1-way ANOVA. (**E**) Schematic depicting generation of optoIDR constructs. mCh indicates mCherry fluorophone and Cry2 encodes for *Arabidopsis thaliana* protein with light-activated phase separation characteristics. (**F**) Various domains of CRYAB with localization of serine 59 and arginine 120 residues depicted. (**G**) Representative time-lapse images at *t* = 0 seconds, 100 seconds, 200 seconds, and 300 seconds after light activation in HEK293A cells transfected with constructs generated with CRYAB WT, its phosphorylation-deficient mutant (S59A), phospho-mimetic mutant (S59D), R120G mutant, or the R120G and S59A double-mutant proteins as the IDR in the optoIDR constructs. Cry2 fused with mCherry without an IDR was used as the negative control, and FUS-N fused with mCherry-Cry2 was studied as positive control. (**H**) Average number of condensates/cell at *t* = 0 versus *t* = 300 seconds in cells treated as in **E**. **P < 0.01 by Mann-Whitney test. (**I**) Average area of condensates/cell at *t* = 300 seconds in cells treated in **E**. ***P < 0.001; ****P < 0.0001 by Tukey’s post hoc test after 1-way ANOVA.

To examine the functional relevance of serine 59 phosphorylation, we generated CRYAB mutants that mimic a phosphorylation-deficient state at that residue by replacing serine with alanine (S59A) or a phosphomimetic state with a change to aspartic acid (S59D). We also generated the CRYAB-R120G mutant with the S59A change and expressed these mutants with an N-terminal GFP tag in HEK293 cells to examine the relevance of serine 59 phosphorylation in regulating its aggregation potential. Despite being expressed at equivalent levels (Figure 2A), the S59D change resulted in formation of GFP-positive CRYAB aggregates, mimicking the observations with the R120G mutant (Figure 2, B and C). By contrast, the S59A change markedly reduced the aggregation of the CRYAB-R120G mutant protein, indicating that serine 59 phosphorylation is necessary for its aggregate-prone behavior (Figure 2, B and C). As we have previously demonstrated (18), aggregate-prone CRYAB-R120G was toxic and induced cell death (Figure 2D). The S59D mutant was indeed sufficient to induce increased cytotoxicity as compared with WT CRYAB, whereas the S59A mutant attenuated the toxicity of the CRYAB-R120G mutant (to a similar extent as observed with CRYAB triple mutant with serine-to-alanine change at 19, 45 and 59; Supplemental Figure 4, A–C), paralleling the observations with their aggregate-prone behavior (Figure 2D).

### Phosphorylation at serine 59 alters the phase-separation behavior of CRYAB.

An emerging body of evidence indicates that phase separation of proteins regulates their ability to form biomolecular assemblies termed as condensates, and the biophysical properties that determine the dynamicity and fluidity of condensates regulate their aggregation potential (24, 26, 28). Accordingly, to examine whether CRYAB can phase separately in living cells, we adapted the OptoDroplet assay system (39) and expressed full-length CRYAB, as well as its N-terminus, C-terminus, and α-crystallin domain (ACD), separately (Figure 2, E and F). We employed the N-terminal domain of FUS (a condensate-forming protein implicated in neurodegeneration as a positive control) and a Cry2 construct lacking an IDR as a negative control (39) and examined their propensity to phase separate after light activation. As shown, light activation resulted in dynamic phase separation of CRYAB into condensates, mimicking the observations with the N-terminal fragment of FUS protein, while Cry2 protein by itself did not phase separate, as previously described (39) (Figure 2G and Supplemental Videos 1 and 2). Remarkably, CRYAB full-length protein and each of CRYAB’s N-terminus, C-terminus, and ACD domains demonstrate the propensity to phase separate (Figure 2, G and H, Supplemental Figure 5, and Supplemental Videos 3–6). While some spherical CRYAB condensates were observed at baseline, prior to light activation, the average number of condensates doubled after light activation (Figure 2, G and H, and Supplemental Video 3). Interestingly, a S59A or S59D change in the full-length protein completely abrogated light-induced condensate formation (Figure 2, G and H, and Supplemental Videos 7 and 8). However, like the observations with GFP-tagged proteins (Figure 2, A–C), the mCherry-tagged optodroplet (optoIDR) constructs induced protein aggregates in cells expressing S59D and CRYAB-R120G mutant proteins (Figure 2, G–I, and Supplemental Videos 8 and 9) even prior to light activation, with markedly larger and irregularly shaped protein aggregates in S59D-transfected cells mimicking the R120G mutant (Figure 2, G and I). In agreement with the observations with the GFP-tagged CRYAB-R120G-S59A versus the CRYAB-R120G constructs (Figure 2, A–C), the S59A change reduced the size of R120G optoDroplet construct aggregates (Figure 2, G and I, and Supplemental Video 10), but did not result in a noticeable light-induced increase in condensate formation (Figure 2, G and H).

**Figure 3:**
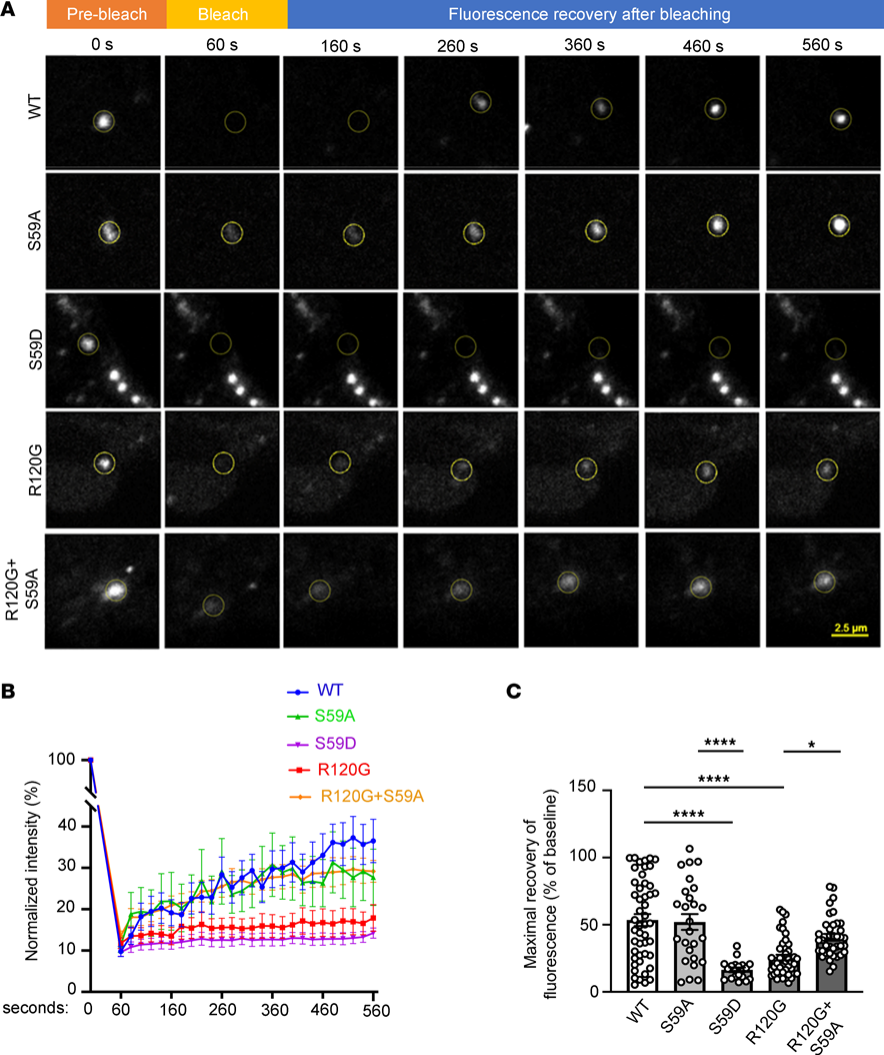
Phosphorylation of CRYAB at serine 59 reduces dynamicity of condensates. (**A**) Representative images demonstrating recovery of fluorescence after photobleaching in HEK 293A cells transfected with mCherry-Cry2 fused optoIDR constructs generated with CRYAB WT, its phosphorylation-deficient mutant (S59A), phospho-mimetic mutant (S59D), R120G mutant, or the R120G and S59A double-mutant proteins. Representative images demonstrate area of photobleaching (marked with a dotted circle) prior to (prebleach), immediately after, and at 100, 200, 300, 400 and 500 seconds after photobleaching was terminated. Intensity at various time points is depicted as a fraction of intensity prior to bleaching (set at 100%). (**B** and **C**) Fluorescence intensity normalized to baseline (**B**) and quantitation of fluorescence recovery (**C**, maximum minus immediately after bleach) in condensates of various CRYAB variants indicated in **A**. *P < 0.05; ****P < 0.0001 by Tukey’s post hoc test after 1-way ANOVA.

These findings suggest that, similar to the R120G mutation, the S59D change reduces the fluidity of condensates, making CRYAB aggregate prone. To examine this premise, we performed fluorescence recovery after photobleaching (FRAP). As shown in Figure 3, A–C, WT CRYAB shows rapid recovery following photobleaching, indicating that these phase-separated condensates are dynamic and liquid like. In contrast, the recovery of fluorescence was markedly reduced in S59D and R120G mutant proteins as compared with WT CRYAB (Figure 3, A–C). Remarkably, the S59A change in R120G restored its fluorescent recovery to WT levels (Figure 3, A–C). The fluorescence recovery was comparable in the S59A mutant to WT CRYAB (Figure 3, A–C). Taken together, these data indicate that serine 59 phosphorylation is both necessary and sufficient to alter the phase separation behavior of CRYAB, reducing fluidity of CRYAB condensates to convert them to a gel-like state (25, 26) as a potential explanation for formation of aggregates.

### Mice bearing phosphomimetic aspartic acid residue in CRYAB at position 59 demonstrate protein aggregates in the myocardium.

**Figure 4:**
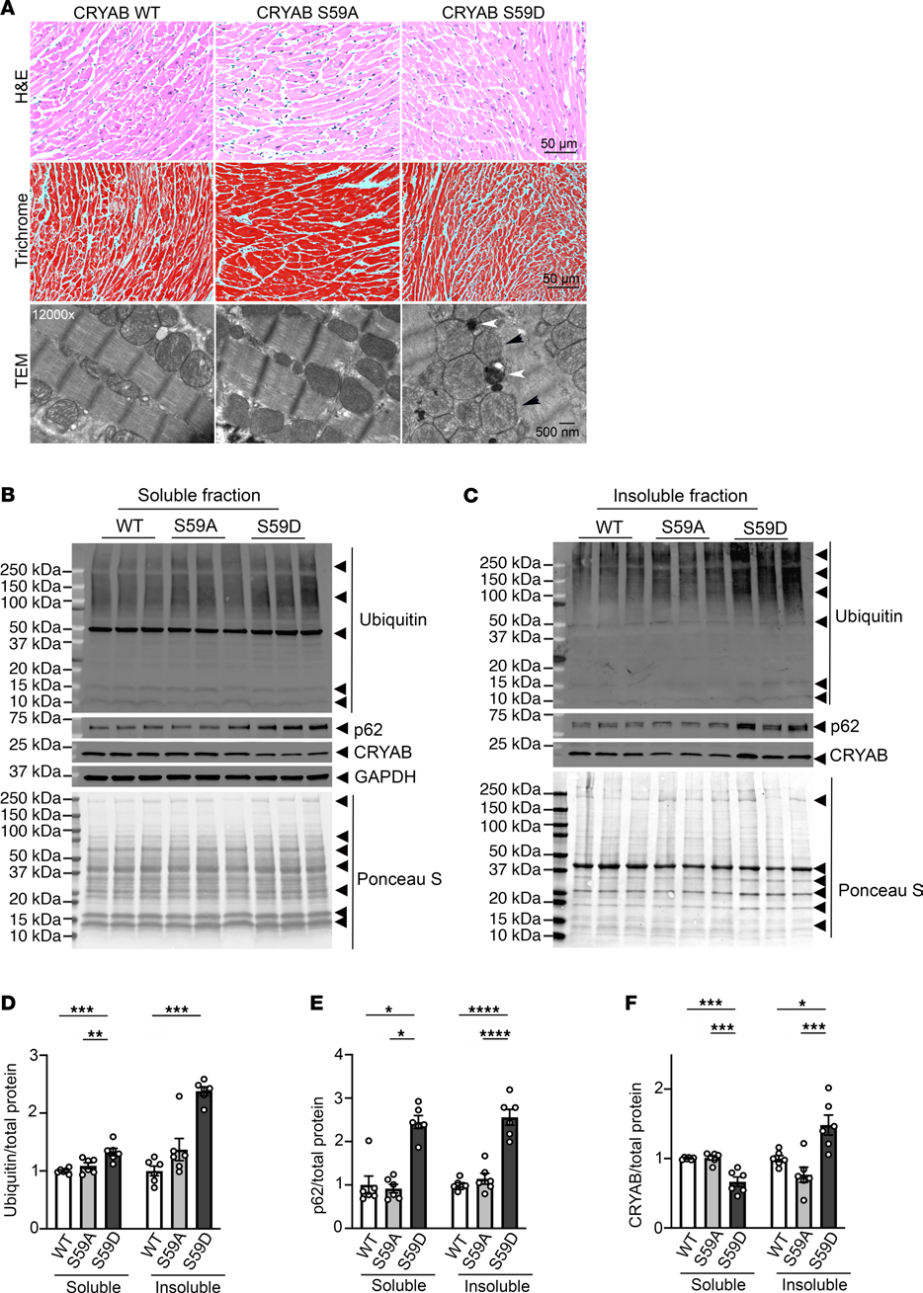
Mice with phosphomimetic aspartic acid residue instead of serine at position 59 in CRYAB demonstrate myocardial protein aggregates. (**A**) Representative H&E- (top row) and Masson’s trichrome–stained (middle row) myocardial sections from young adult mice homozygous for alleles bearing serine–to–aspartic acid (phosphomimetic residue; S59D) or serine-to-alanine (phosphorylation deficient; S59A) mutation at position 59 in CRYAB and mice bearing WT CRYAB alleles as controls. Transmission electron micrographs (bottom row) from WT, S59A, and S59D mice. Representative of *n* = 2 mice per group. Black arrowheads point to abnormal-appearing mitochondria and white arrowheads point to protein aggregates. (**B** and **C**) Representative immunoblots depicting expression of ubiquitinated proteins and p62, CRYAB, and GAPDH proteins in NP40-soluble (**B**) and NP40-insoluble (**C**) myocardial extracts from WT, S59A, and S59D mice. Ponceau S staining is shown as loading control. (**D**–**F**) Quantitation of ubiquitinated proteins (**D**), p62 (**E**), and CRYAB (**F**) in soluble and insoluble myocardial extracts from WT, S59A, and S59D mice. *P < 0.05; **P < 0.01; ***P < 0.001; ****P < 0.0001 by Tukey’s post hoc test after 1-way ANOVA, except for the data in the insoluble fraction in **D** and soluble fraction in **E**, which were analyzed by Dunn’s multiple-comparison test after Kruskal-Wallis test.

To rigorously determine the role of phosphorylation of the serine 59 residue of CRYAB in myocardial homeostasis and response to stress, we used CRISPR/Cas9 editing to generate mice homozygous for phosphorylation-deficient serine-to-alanine change (S59A) and phosphomimetic serine–to–aspartic acid change (S59D). We confirmed the loss of immunodetectable pS59 CRYAB in the S59A myocardium (Supplemental Figure 6). Either amino acid substitution did not alter total CRYAB levels (Supplemental Figure 7), left ventricular structure and function, or body weight (Supplemental Table 2) or result in myocardial histologic abnormalities in young adult mice of both genotypes (Figure 4A), which were also grossly indistinguishable from WT mice. However, transmission electron microscopy revealed abnormal mitochondria with cristal rarefaction and presence of protein aggregates in cardiac myocytes from S59D mice (Figure 4A). The myocardium in S59A mice was ultrastructurally indistinguishable from that of WT mice (Figure 4A). This was correlated with biochemical evidence of increased ubiquitinated proteins (Figure 4, C and D), including an increase in polyubiquitinated proteins (Supplemental Figure 8), in the aggregate-rich insoluble fraction in S59D mice as compared with WT and increased p62 in both soluble and insoluble fractions (Figure 4, C and E) in myocardium from S59D mice as compared with WT and S59A mice. Notably, there was a reduction in CRYAB protein abundance in the soluble fraction from S59D mice and a concomitant increase in the aggregate-rich insoluble fraction as compared with the other 2 genotypes (Figure 4, B, C, and F), pointing to increased propensity of S59D CRYAB to form protein aggregates as we have observed earlier (Figure 2, A–C). Mice bearing phosphorylation-deficient serine-to-alanine change in CRYAB were indistinguishable from WT on the above parameters.

### Phosphorylation-deficient serine-to-alanine change in CRYAB at position 59 attenuates post-MI left ventricular systolic dysfunction.

**Figure 5:**
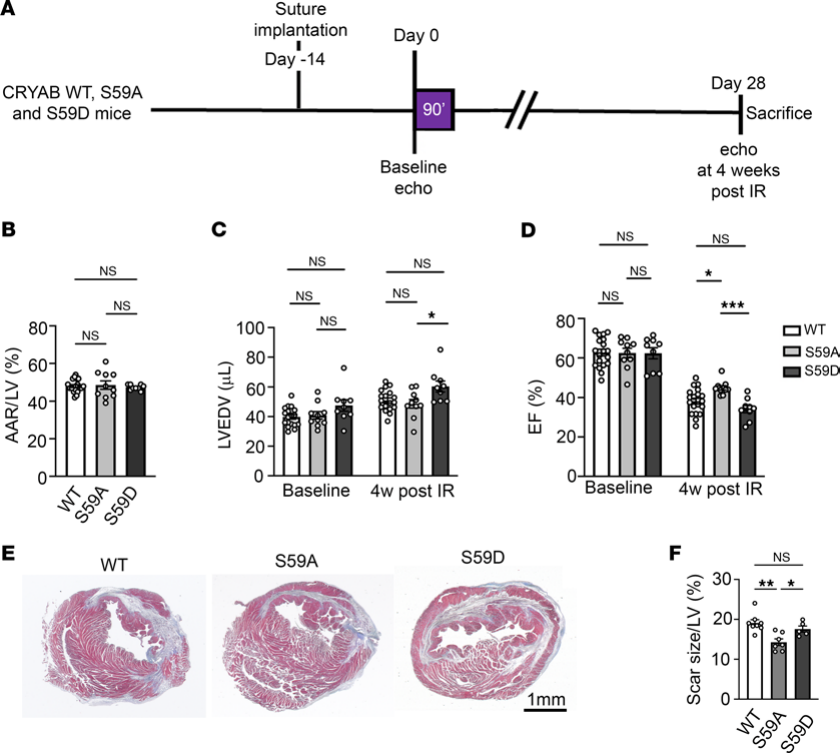
Knockin of phosphorylation-deficient alanine instead of serine at position 59 in CRYAB rescues post-IR left ventricular remodeling in mice. (**A**) Schematic depicting experimental strategy for closed-chest IR modeling (90 minutes of ischemia followed by reperfusion) in S59A, S59D, and WT. (**B**) Quantitative assessment of area-at-risk (AAR) during LAD occlusion in mice treated as in **A**. (**C** and **D**) Quantitative analyses of left ventricular EDV (LVEDV, **C**) and LV EF (EF (%), **D**) at baseline (i.e. prior to) and at 4 weeks after IR injury. *P < 0.05; ***P < 0.001 by Tukey’s post hoc testing after 1-way ANOVA. Echocardiographic parameters at baseline and 4 weeks after IR injury were analyzed separately as they were performed under different anesthetic regimens. (**E** and **F**) Masson’s trichrome–stained left ventricular sections demonstrating presence of scar at 4 weeks after IR injury (**E**) with quantitation of scar size (**F**). *P < 0.05; **P < 0.01 by Tukey’s post hoc testing after 1-way ANOVA.

To test the hypothesis that stress-induced phosphorylation of serine 59 in CRYAB in the post-MI myocardium is a mechanism for adverse left ventricular remodeling (as observed in Supplemental Figure 2), we subjected young adult S59A and S59D mice and WT controls to closed chest IR modeling and examined left ventricular structure, function, and scar size at 4 weeks after MI (Figure 5A and Supplemental Table 3). Mice bearing S59A CRYAB alleles demonstrated improved left ventricular EF at 4 weeks after MI as compared with WT mice and with mice bearing S59D alleles (Figure 5D) despite comparable area at risk (Figure 5B). Also, left ventricular dilation after MI was significantly reduced in S59A versus S59D mice (Figure 5C) indicating that preventing phosphorylation at serine 59 abrogated adverse left ventricular remodeling after MI. This was also accompanied by reduced scar size in the S59A mice as compared with the other 2 genotypes (Figure 5, E and F), which was not due to a reduction in infarct size in S59A versus WT at 24 hours after injury (Supplemental Figure 9), indicating prevention of infarct expansion. Taken together, these data indicate that phosphorylation as serine 59 contributes to adverse left ventricular remodeling after MI.

### Phosphorylation of CRYAB at serine 59 increases its interaction with desmin, resulting in its mislocalization to protein aggregates.

**Figure 6:**
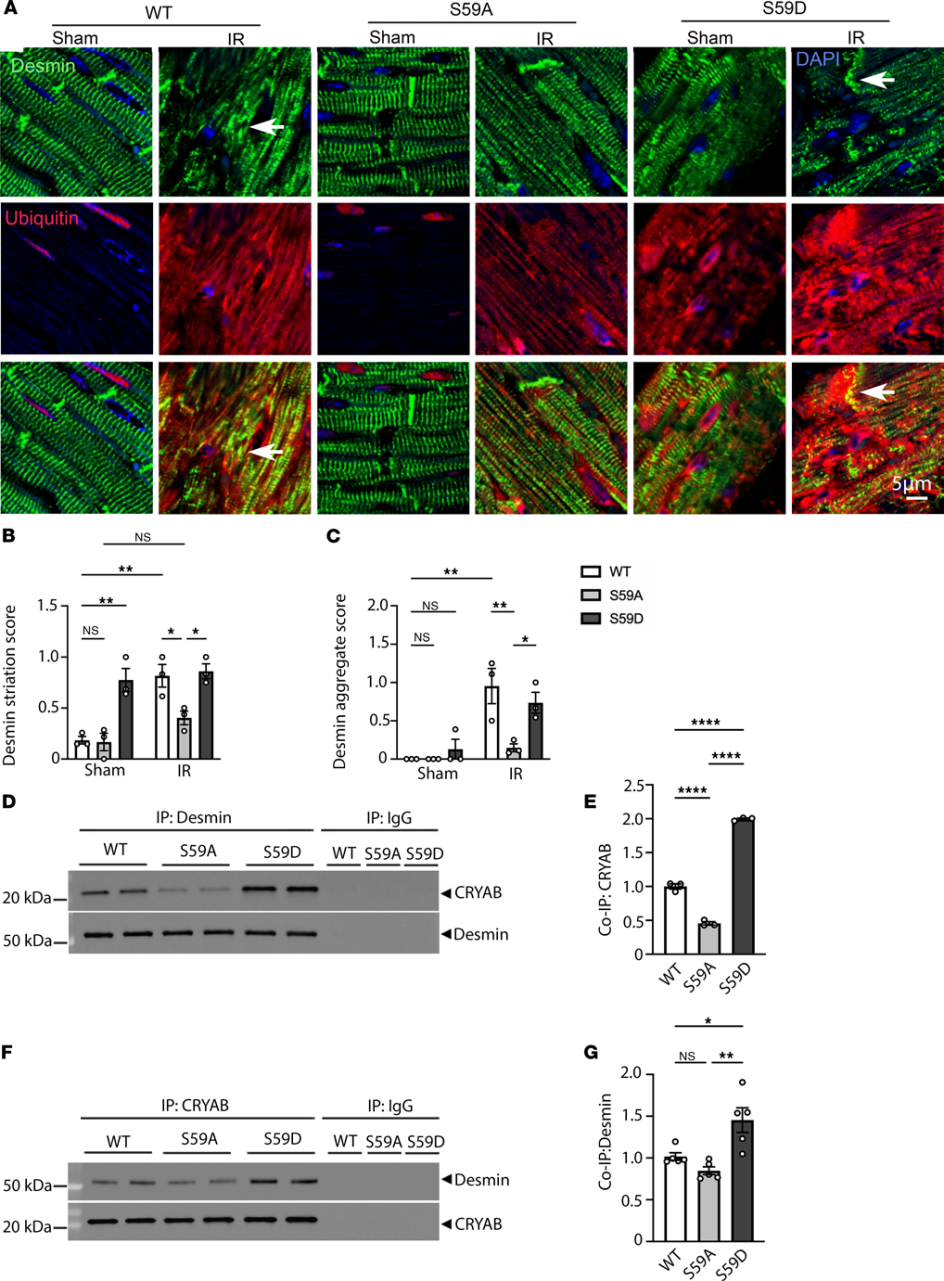
Knockin of phosphorylation-deficient alanine or phosphomimetic aspartic acid instead of serine at position 59 in CRYAB has opposing effects on desmin localization. (**A**) Representative immunohistochemical images demonstrating localization of desmin (top row) and ubiquitinated proteins (middle row) and merged images (bottom row) from S59A, S59D, and WT mice 4 weeks after being subjected to closed-chest IR injury or sham procedure. Arrows point to desmin aggregates. (**B** and **C**) Quantitative evaluation of desmin localization with striation score (**B**) and aggregated desmin (**C**) in S59A, S59D, and WT mice 4 weeks after being subjected to closed-chest IR injury or sham procedure. For striation scoring, normal localization of proteins got scored as 0, and abnormal striation or mislocalization of proteins was scored as of 1. For scoring aggregates, absence of aggregates was scored as 0 and presence of aggregates was scored as 2. *P < 0.05; **P < 0.01 by Tukey’s post hoc testing after 1-way ANOVA. (**D** and **E**) Representative immunoblot (**D**) and quantitative assessment (**E**) of CRYAB and desmin expression after IP with desmin from myocardial extracts from young adult S59A, S59D, and WT mice. Expression of CRYAB is assessed as fold of WT control. (**F** and **G**) Representative immunoblot (**F**) and quantitative assessment (**G**) of CRYAB and desmin expression after IP with CRYAB from myocardial extracts from young adult S59A, S59D, and WT mice. Expression of desmin is assessed as fold of WT control. *P < 0.05; **P < 0.01; ****P < 0.0001 by Tukey’s post hoc testing after 1-way ANOVA.

We next examined the effect of CRYAB phosphorylation on its interaction with its client protein desmin in the myocardium using mice modeled to generate phosphorylation-deficient (S59A) and phosphomimetic (S59D) changes in CRYAB. Desmin was mislocalized from striations with increased aggregates at 4 weeks after IR injury with increase in ubiquitinated proteins in the myocardium as compared with sham controls (Figure 6, A–C). Interestingly, as compared with WT in the post-MI myocardium, normal desmin localization to striations was better preserved in S59A with reduced desmin aggregates (Figure 6, A–C). In contrast, desmin was mislocalized with increased aggregates even in sham-treated S59D myocardium and this persisted at 4 weeks after MI (Figure 6, A–C). A potential explanation for these findings is the increased interaction between CRYAB phosphorylated at serine 59 with desmin. Indeed, we have previously observed that R120G mutant CRYAB (which is predominantly phosphorylated at serine 59 residue, Supplemental Figure 3) demonstrates increased interaction with desmin as compared with WT CRYAB (18). Accordingly, we performed immunoprecipitation (IP) studies to examine the interaction between CRYAB and desmin in the myocardium from unstressed young adult S59A, S59D, and WT mice and observed increased interaction between desmin and S59D as compared with WT CRYAB and reduced interaction between desmin and S59A CRYAB as compared with WT CRYAB (Figure 6, D–G), a finding that was confirmed by reverse co-IP studies as shown. Taken together, these findings suggest that phosphorylation of CRYAB at serine 59 alters its condensate properties to make it aggregate prone and increases its binding with client proteins as the mechanism for mislocalization of these proteins (such as desmin, α-actinin, and actin as in Figure 1A) to aggregates.

### Attenuating serine 59 phosphorylation with 25-HC rescues post-IR cardiomyopathy.

**Figure 7:**
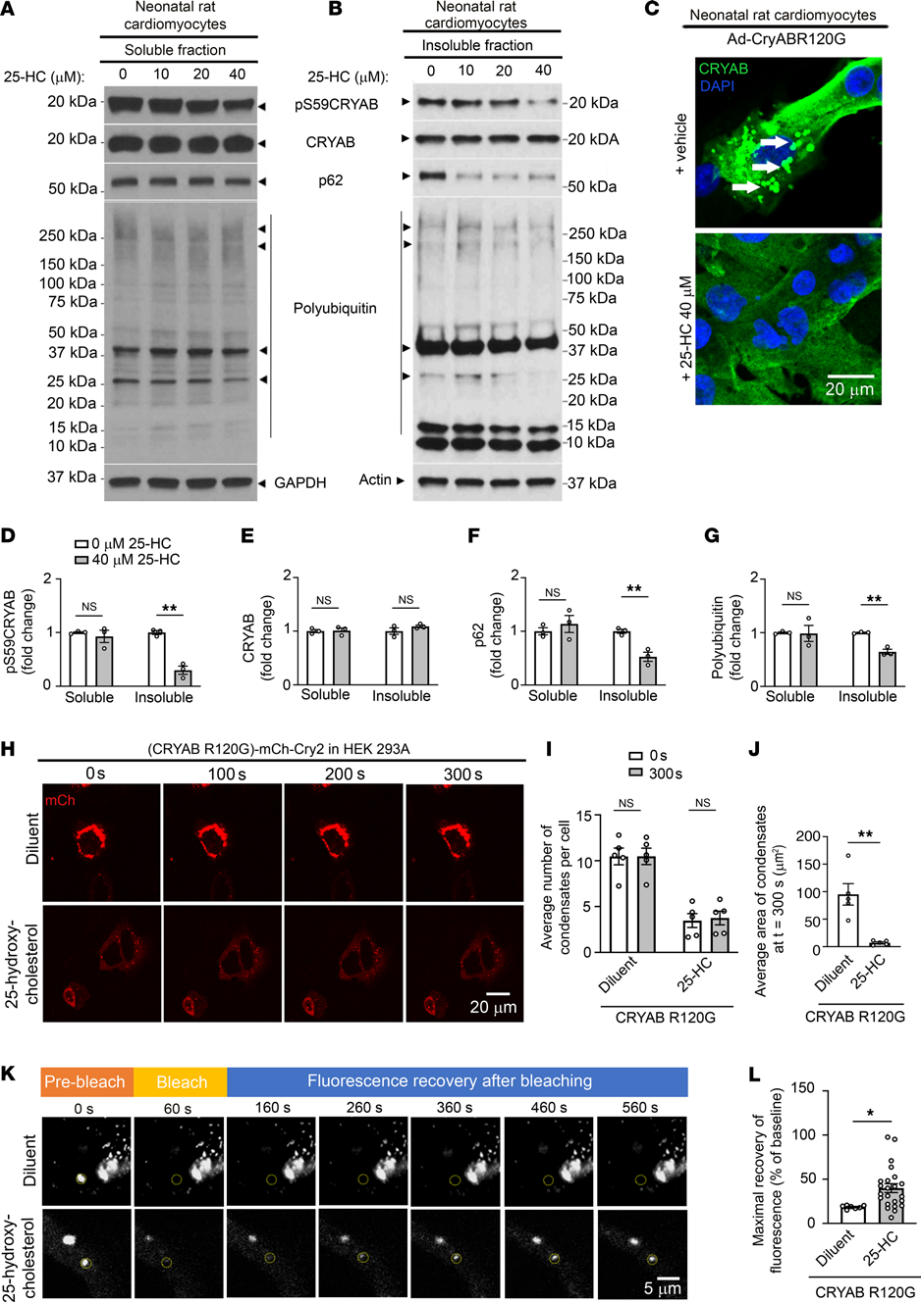
Treatment with 25-HC reduces phosphorylation of CRYAB at S59 and alters the phase-separation characteristics of CRYAB-R120G. (**A** and **B**) Representative immunoblots depicting total pS59-CRYAB, CRYAB, p62, and polyUb proteins in NP40-detergent (**A**) soluble and (**B**) insoluble fractions from neonatal rat cardiomyocytes (NRCMs) transduced with adenoviral CRYAB-R120G (MOI=10) for 90 hours and treated with 0, 10, 20, and 40 mM 25-HC for the final 72 hours. GAPDH and actin are shown as loading controls. (**C**) Representative immunofluorescence images of NRCMs treated in **A**. Arrows point to GFP-positive aggregates. (**D**–**G**) Quantitative assessment of pS59-CRYAB (**D**), total CRYAB (**E**), p62 (**F**), and polyUb protein (**G**) expression in NP-40 soluble and insoluble fractions from NRCMs treated in **A**. **P < 0.01 by *t* test. (**H**) Representative time-lapse images at *t* = 0 seconds, 100 seconds, 200 seconds, and 300 seconds after light activation in HEK293A cells transfected with OptoIDR constructs generated with CRYAB-R120G as the IDR protein and treated with diluent or 25-HC (40 mM). (**I** and **J**) Average number (**I**) and area (**J**) of condensates/cell at *t* = 0 versus *t* = 300 seconds in cells treated as in **H**. **P < 0.01 by *t* test. (**K**) Representative images demonstrating recovery of fluorescence after photobleaching in HEK 293A cells treated as in **H**. The area of photobleaching is marked with a dotted circle prior to (prebleach), and at 0, 100, 200, 300, 400, and 500 seconds (s) after photobleaching. (**L**) Quantitation of fluorescence recovery in condensates of CRYAB variants (as maximum minus immediately after photobleaching) as shown in **K**. *P < 0.05 by *t* test.

Our data indicate that pS59-CRYAB is a toxic aggregate-prone protein and a phosphorylation-deficient mutant of CRYAB attenuates post-MI adverse left ventricular remodeling. To examine the paradigm that a pharmacologically induced reduction in pS59-CRYAB may be beneficial in the post-MI setting, we turned to studies with 25-HC. This compound was uncovered in a screen focused on preventing aggregation of CRYAB-R120G in the lens of the eye to prevent cataract formation (27). It has been predicted to bind in a groove in CRYAB (40), whereby we hypothesized that it will prevent phosphorylation of CRYAB, perhaps by restricting access to serine 59. Accordingly, we tested the efficacy of 25-HC on serine 59 phosphorylation of CRYAB-R120G protein transduced in neonatal rat cardiac myocytes, followed by subcellular fractionation. As shown, 25-HC induced a dose-dependent reduction in pS59-CRYAB levels in the detergent-insoluble fraction (Figure 7B) with a significant reduction at the highest dose tested (Figure 7, B and D) without a change in its levels in the soluble fraction (Figure 7, A and D) or in total CRYAB abundance (Figure 7, A, B, and E). This was accompanied by a reduction in p62 and polyUb proteins in the insoluble fraction (Figure 7, B, F, and G) but not the soluble fraction (Figure 7, A, F, and G) with 40 μM 25-HC. 25-HC also induced a marked reduction in CRYAB-R120G aggregates detected with immunostaining (Figure 7C). To determine whether 25-HC treatment alters phase separation of the CRYAB-R120G mutant protein, we expressed CRYAB-R120G optoIDR construct (as in Figure 2, E–G) and treated the cells with 40 μM 25ΗC or diluent. 25-HC treatment reduced the number and size of CRYAB-R120G aggregates (Figure 7, H and J), but did not result in a noticeable light-induced increase in condensate formation (Figure 7, H and I, Supplemental Videos 11 and 12). To examine the effect of 25-HC treatment on the fluidity of CRYAB-R120G condensates, we performed FRAP analyses. As shown (Figure 7, K and L), the recovery of fluorescence was markedly increased in 25-HC–treated CRYAB-R120G mutant protein after bleach as compared with diluent-treated CRYAB-R120G. Importantly, 25-HC did not affect condensate number or size or the fluidity of S59D CRYAB condensates (Supplemental Figure 10, Supplemental Videos 13 and 14). Taken together, these data indicate that 25-HC treatment reduces serine 59 phosphorylation to maintain fluidity of the resulting condensates and reduces the propensity for aggregate formation.

**Figure 8:**
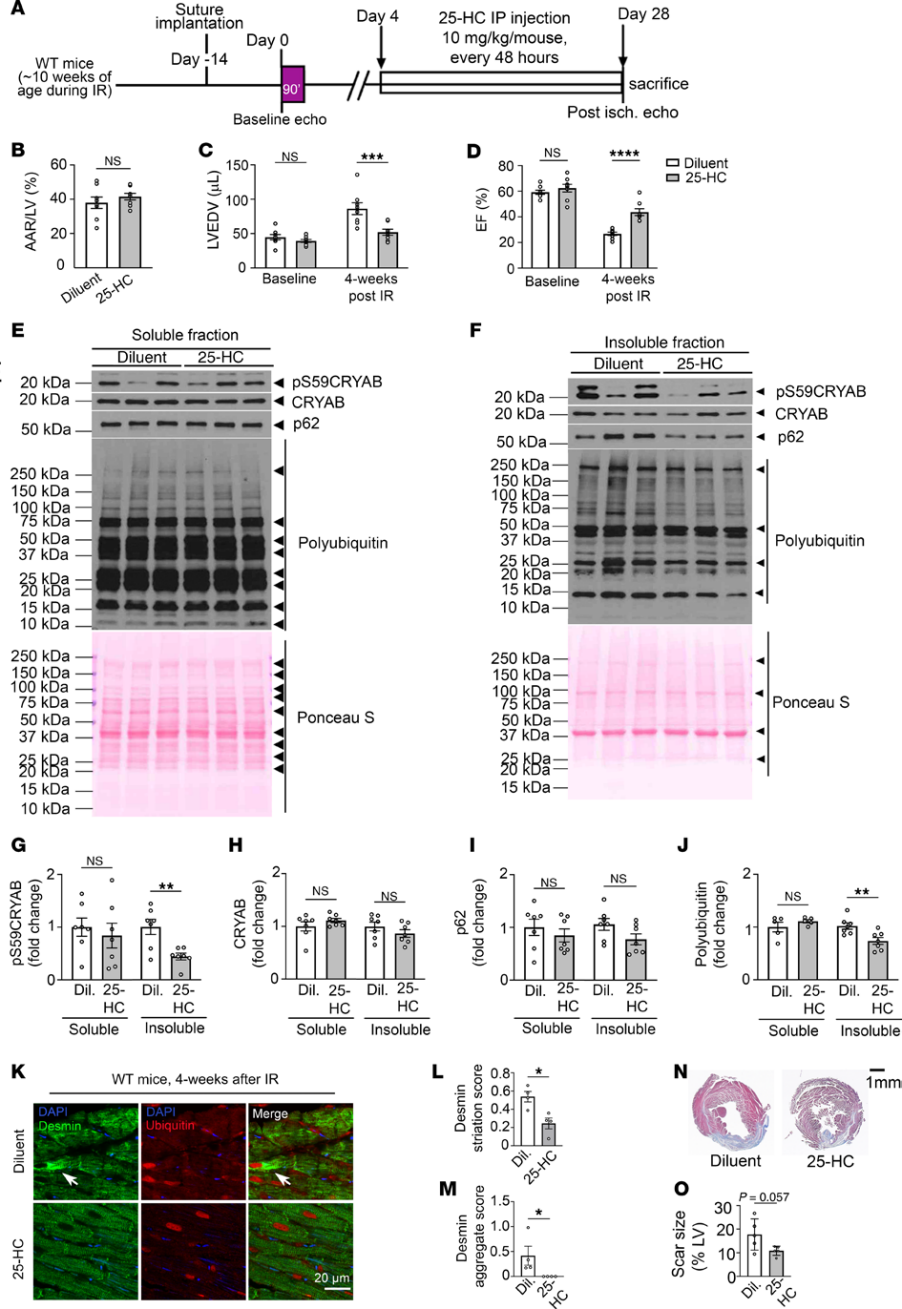
Treatment with 25-HC rescues adverse left ventricular remodeling after IR injury. (**A**) Schematic depicting experimental strategy for closed-chest IR modeling (90 minutes of ischemia followed by reperfusion) in male WT mice followed by intraperitoneal administration of 25-HC (10 mg/kg/mouse, every 48 hours) or diluent, initiated on day 4 after IR injury. (**B**–**D**) Quantitative analyses of area-at-risk (AAR, **B**), left ventricular EDV (LVEDV, **C**) and LV EF (EF) (%), **D**) prior to and at 4 weeks after IR injury in mice treated as in **A**. ***P < 0.001; ****P < 0.0001 by *t* test. (**E** and **F**) Immunoblots depicting total CRYAB, pS59-CRYAB, p62, and polyUb protein expression in NP-40 soluble (**E**) and insoluble (**F**) fraction in myocardium of mice 4 weeks after IR injury and treated with diluent or 25-HC. (**G**–**J**). Quantitative analyses of pS59-CRYAB (**G**), total CRYAB (**H**), p62 (**I**), and polyUb proteins (**J**) in NP-40 soluble and insoluble fraction in myocardium of mice 4 weeks after IR injury and treated with diluent or 25-HC. **P < 0.01 by *t* test. (**K**) Representative images with immunofluorescence staining for desmin and ubiquitinated proteins in the remote myocardium from mice modeled as in **A**. (**L** and **M**) Quantitative evaluation of desmin localization with striation score (**L**) and aggregated desmin (**M**) in mice treated as in **A**. For striation scoring, normal localization of proteins got scored as 0, and abnormal striation or mislocalization of proteins was scored as 1. For scoring aggregates, absence of aggregates was scored as 0, and presence of aggregates was scored as 2. *P < 0.05 by *t* test. (**N** and **O**) Masson’s trichrome–stained left ventricular sections (**N**) demonstrating presence of scar at 4 weeks after IR injury in mice treated as in **A** with quantitation of scar size (**O**). *P* value shown is by *t* test.

These findings predict a beneficial effect of 25-HC on post-IR remodeling by reducing the abundance of pS59-CRYAB. Accordingly, we set up a protocol where mice were injected with 25-HC at the dose of 10 mg/kg beginning at day 4 after IR injury (Figure 8A). We chose this time point to avoid its effects on CRYAB phosphorylation during the acute phase of injury, where p38 MAPK–mediated serine 59 phosphorylation may play a protective role (41, 42). Our data (Supplemental Table 4) demonstrate a significant increase in left ventricular ejection fraction (Figure 8D) with reduced left ventricular dilation (Figure 8C) in 25-HC–treated mice, while the area at risk was similar between the 2 groups (Figure 8B). We examined the effect of 25-HC on pS59-CRYAB and detected a significant decline in total pS59-CRYAB abundance (Supplemental Figure 11). This was accompanied by a decline in pS59-CRYAB levels in the insoluble fraction in mice treated with 25-HC (by 56%, Figure 8, F–H) without a change in the soluble fraction (Figure 8, E and G), consistent with the effects observed in neonatal rat cardiac myocytes (Figure 7, A, B, and D). Interestingly, 25-HC treatment also modestly reduced the abundance of pS59-CRYAB in both soluble and insoluble myocardial fractions from sham-operated mice (Supplemental Figure 12). Notably, 25-HC treatment induced a decline in polyUb proteins but not p62 in the insoluble fraction (Figure 8, F, I, and J). 25-HC treatment also restored physiologic desmin localization and attenuated aggregates with reduced ubiquitinated proteins observed in the post-IR myocardium of WT mice (Figure 8, K–M). There was no significant difference in scar size in 25-HC versus diluent-treated mice after MI (Figure 8, N and O). These data support the notion that attenuating overall levels of serine 59–phosphorylated CRYAB is an effective strategy to reduce the aggregation potential and toxicity of CRYAB under sustained stress, as observed in ICM.

## Discussion

**Figure 9:**
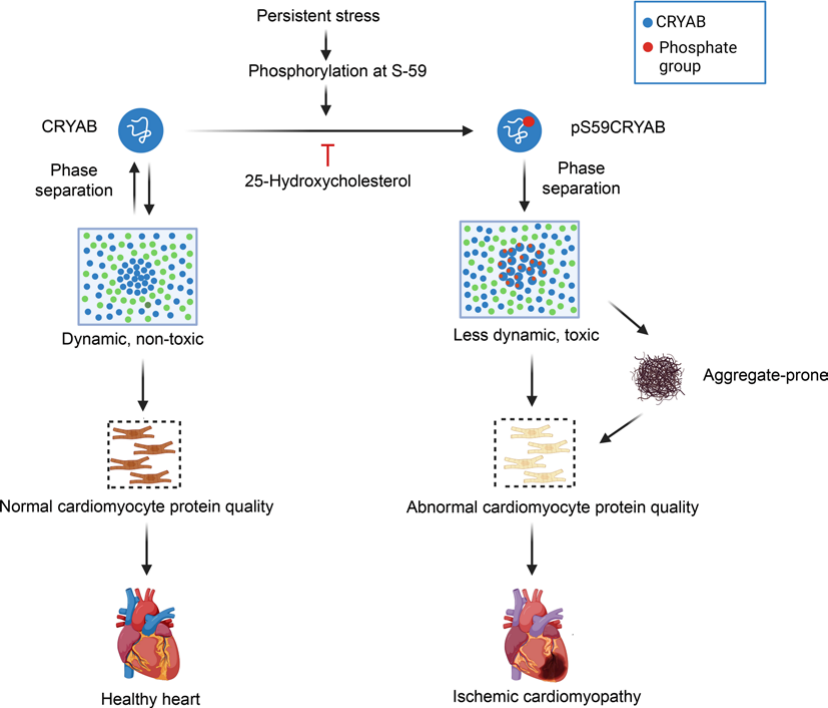
Schematic depicting consequences of serine 59 phosphorylation on phase separation of CRYAB in ICM. CRYAB undergoes dynamic phase separation into condensates in physiology with maintenance of cardiac myocyte health in homeostasis. Persistent stress, as observed with myocardial IR injury (or with human disease-causing R120G mutation in CRYAB protein, not shown), results in phosphorylation at serine 59. Phosphorylated CRYAB at serine 59 (pS59-CRYAB) undergoes phase separation into condensates with reduced dynamicity and increased toxicity. pS59-CRYAB partitions into the aggregate-rich NP-40 insoluble fraction with segregation of its client proteins, such as desmin, α-actinin, and actin, in aggregates within cardiac myocytes in ICM. Inhibiting phosphorylation of CRYAB at serine 59 prevents it from becoming aggregate-prone and attenuates development of cardiomyopathy in the setting of IR injury. Image prepared with BioRender software.

Protein aggregates are a hallmark of neurodegenerative diseases and are implicated in pathogenesis of rare genetic and amyloid cardiomyopathies. Our work demonstrates a mechanistic role for protein aggregation in the pathogenesis of ICM, a prevalent condition which is the leading cause of heart failure worldwide. These findings implicate a broader pathogenic role for condensatopathy as the underlying mechanism in cardiac disease beyond that observed in rare genetic cardiomyopathy, such as the condition provoked by a mutant RBM20 protein (43). A mechanistic framework that emerges is that CRYAB, a highly enriched chaperone protein in cardiac myocytes, undergoes phase separation into liquid-like dynamic condensates under physiologic conditions (Figure 9). Sustained stress, as with development of ICM, results in increased phosphorylation of CRYAB at serine 59 (Figure 1 and Supplemental Figure 2), which alters its phase-separation behavior (Figure 2 and Figure 3). Phosphomimetic serine–to–aspartic acid change at position serine 59 in CRYAB induces gel-like condensates with reduced dynamicity and increased interaction with clients such as desmin, triggering aggregate formation and cytotoxicity and sequestration of desmin in protein aggregates (Figures 4–6). Mice harboring nonphosphorylatable alanine instead of serine at position 59 in CRYAB are partially protected from post-MI left ventricular systolic dysfunction and desmin mislocalization with reduced scar size at 4 weeks after MI (Figure 5 and Figure 6). In addition, pharmacologic reduction of the overall abundance of pS59-CRYAB by 25-HC treatment after MI reduces protein aggregates with preserved desmin localization and attenuates ICM (Supplemental Figures 7–9). These findings underscore the notion that targeting the mechanisms of altered phase separation, i.e., abnormal condensate behavior of CRYAB, is a clinically translatable strategy to ameliorate pathology in ICM.

Phase separation of proteins (along with other biomolecules) and their regulation by posttranslational modifications has emerged as a critical mechanism underlying the control of spatiotemporal organization of cellular components in physiology (44, 45). Cellular structures, such as P-bodies and stress granules, are formed by liquid-liquid phase separation and retain dynamic flux across the boundaries to facilitate rapid formation and reversibility (45). Interestingly, long-lasting subcellular structures, such as the nucleolus and centromeres that are less dynamic, are also formed and maintained through phase separation and are conceived as more gel-like (46–48). Dysregulation of the physiologic role of biomolecular condensates has been implicated in the pathogenesis of cancer and neurodegenerative and infectious diseases (28), but its role in human cardiac pathology has not been widely explored. A carefully performed experimental analysis of the R636S variant of human RNA-binding motif protein-20 (RBM20) that has been causally linked to development of dilated cardiomyopathy, revealed dysregulation of RNA-protein condensates in the cytosol as the pathogenic mechanism (43). Recent experimental studies identified that HIP-55 (hematopoietic progenitor kinase 1–interacting protein of 55 kDa), a negative regulator of β-adrenoceptor signaling, undergoes phase separation to form condensates; and reduced phosphorylation dysregulates HIP-55 condensates to its aggregation and cardiac dysfunction in mice challenged with isoproterenol (49). Our observation of CRYAB phosphorylation-induced condensatopathy after MI is the first, to our knowledge, to implicate a role for dysregulated condensates in a common etiology for heart failure.

Our data suggest that reversible stress-induced phosphorylation of CRYAB is likely a mechanism to regulate protein-protein interactions with client proteins by altering the dynamicity of the resultant condensates and confers cytoprotection or cytotoxicity, depending upon the cellular context. Under acute stress, p38 kinase activation and resultant serine 59 phosphorylation of CRYAB is likely to be temporally limited and reversible with inactivation of p38 when stress abates, facilitating increased interaction with its client proteins such as desmin by reducing dynamicity of condensates, as a protective mechanism only while the stress lasts, akin to the physiology observed with stress granules (44). Indeed, CRYAB is rapidly phosphorylated at serine 59 with ischemia and localizes to the Z-discs in cardiac myocytes (31, 50) where it interacts with desmin with increased affinity under low pH (31) and binds tightly to its other client proteins including Titin, in a manner resistant to solubilization with urea (51). Also, CRYAB was noted to be transcriptionally upregulated in differentiating neural progenitor cells and localizes to aggregates to sequester misfolded proteins; and siRNA-mediated knockdown of CRYAB prevents sequestration of misfolded proteins with reduced cell viability (52). Analogously, overexpression of CRYAB with a phosphomimetic serine–to–aspartic acid change at position 59 conferred cytoprotection against hypoxic stress in isolated cardiac myocytes (41). In contrast, persistent stress with development of ICM induces sustained serine 59 phosphorylation with persistent p38 kinase activation (37), whereby the pS59-CRYAB becomes a “toxic” protein and requires sequestration within aggregates to mitigate toxicity. Indeed, prior studies have shown a high propensity for pS59-CRYAB to partition in a detergent-insoluble fraction in aggregates, termed Rosenthal bodies, in brains of patients with Alexander’s disease and in Alzheimer’s disease (53), in the setting of persistent disease pathology.

The propensity of CRYAB to phase separate through intermolecular interactions to form higher order structures is likely to be facilitated by its IDRs, consistent with the reported heterogeneity of CRYAB multimers ranging from 10–40 subunits (54), indicating its polydisperse nature (55). Also, in rigorous biophysical studies, the N-terminal region of human CRYAB (residues 1–67) was found to exist in different states consistent with the notion of being intrinsically disordered and to contribute to the observed heterogeneity of the CRYAB protein multimers (56). These studies also predict a likely effect of S59 phosphorylation via interactions of the β2 region (Ser59-Thr63) with other regions of CRYAB, both intramolecularly as well as intermolecularly (56) on the composition of CRYAB multimers. Intriguingly, we have observed that 25-HC inhibits phosphorylation of CRYAB at serine 59 as a novel mechanism to prevent aggregation of CRYAB. Our data also uncover this as a novel mechanism of action whereby 25-HC drives increased solubility of the R120G protein with disruption of aggregates, complementary to its ability to bind CRYAB, as suggested by molecular docking studies (40). Indeed, our studies with the phosphorylation-deficient mutant confirm that phosphorylation of CRYAB at serine 59 is essential for formation of aggregates with the R120G mutant. It is also conceivable that 25-HC acts as a hydrotrope to disrupt gel-like condensates of R120G by preventing its multimerization, akin to a role for ATP (57).

Our study is the first, to our knowledge, to mechanistically implicate protein aggregation in the development of ICM. Prior work in myocardial samples from human ICM has demonstrated increased abundance of short fibrils of desmin (~190 kD), which mimic preamyloid oligomers detectable with A11 staining in patients with dilated cardiomyopathy and mice with transgenic expression of R120G mutant CRYAB (16). These studies demonstrate the presence of desmin in protein aggregates in mice subjected to pressure overload (16) or dogs modeled for rapid pacing-induced cardiomyopathy (58); and furthermore, these authors observe that desmin cleavage and phosphorylation are associated with formation of cardiac aggregates and preamyloid oligomers in the observed cardiomyopathy. Our work suggests a mechanism for desmin localization to aggregates by increased interaction with phosphorylated CRYAB, which is aggregate prone (Figure 6). Indeed, other CRYAB client proteins that localize to the sarcomeres that we studied, namely actin and α-actinin, also mislocalize to aggregates in both human and mouse myocardium with ICM, suggesting a general disassembly or misassembly of sarcomeres, a premise that will require experimental validation. It is also conceivable that posttranslational modifications of CRYAB client proteins, such as desmin, also alters their phase-separation properties to drive protein aggregation and cause cardiac dysfunction. Accordingly, preventing aggregate formation or disaggregating protein aggregates may offer a therapeutic opportunity by restoring sarcomere structure and function.

Our discovery of the regulation of phase-separation behavior of a cardiac myocyte–enriched chaperone protein, CRYAB, by a stress-induced posttranslational modification, offers a therapeutic opportunity to explore and harness the beneficial signaling conferred by CRYAB and other chaperone proteins that are critical for cardiac myocyte protein quality control. These observations support a novel paradigm that dysregulation of biomolecular condensates, as observed in rare genetic cardiomyopathies triggered by the R636S mutation in RBM20 (a striated muscle-specific nuclear alternative splicing factor (43)) and with the R120G mutation in CRYAB (vide supra), may be pathogenic in common etiologies of cardiomyopathy, opening the door for targeting biomolecular condensates to develop sarcomere-targeted therapies for highly prevalent causes of heart failure.

## Methods

### Sex as a biological variable.

Mice of both sexes were studied, unless otherwise specified. No significant differences were observed between sexes for the primary phenotype, whereby the data for both sexes were combined for presentation. All observers were blinded.

### Genetically modified mice.

For generating mice with serine-to-alanine change (S59A) and serine–to–aspartic acid change (S59D), we employed CRISPR/Cas9 genome editing. A single active guide RNA (gRNA) was used to introduce point mutations in the CRYAB locus of B6/CBA hybrids using homology-directed repairs (HDR) after the pronuclear introduction of both gRNA and Cas9 was done by electroporation. Electroporation was performed in 4 sessions where 2 different mouse colonies, S59A (AGC to GCC) and S59D (AGC to GAC), were generated with a single electroporation mix (i.e., both single stranded oligodeoxynucleotides [ssODNs] in the same mix). The presence of the CRYAB point mutations was confirmed in each generation of the mouse colony by deep sequencing, and mice from the seventh or later generation of backcrosses into the C57BL/6J strain (JAX strain 000664) were used for the experiments.

### Human heart tissues.

Deidentified frozen heart-tissue samples were obtained from the Human Heart Tissue Bank at the University of Pennsylvania, and formalin-fixed paraffin-embedded left ventricular myocardial tissue was obtained from the Translational Cardiovascular Biobank and Repository at Washington University School of Medicine. The hearts were procured from 2 separate patient groups: nonfailing brain-dead organ donors with no history of heart failure (donor, whose hearts were screened but not selected for transplantation) and heart transplant recipients with advanced ICM, from whom hearts were obtained at the time of orthotopic heart transplantation. Clinical characteristics of the subjects whose human tissue was studied are summarized in Supplemental Table 1.

### Closed-chest cardiac IR modeling.

Mice were subjected to reversible left anterior descending (LAD) coronary artery ligation for 90 minutes followed by reperfusion, in a closed-chest procedure, as described (59). Briefly, mice were anesthetized with ketamine/xylazine (100 mg/kg and 10 mg/kg via intraperitoneal injection), surgically prepped, and ventilated. A left mini thoracotomy was performed, followed by dissection of the pericardium. An 8-0 polypropylene suture was passed under the LAD artery with a U-shaped tapered needle and the 2 ends threaded through a 0.5 mm piece of gas-sterilized PE-10 tubing, forming a loose snare around the LAD artery. The suture ends were exteriorized, the thoracotomy and skin were serially sutured closed, and the mouse allowed to recover. Fourteen days after suture implantation, mice were anesthetized with 1.5% isoflurane with ECG monitoring. The skin above the chest wall was reopened, and the implanted suture ends were pulled apart gently until ST segment elevation confirmed LAD artery occlusion. Following 90 minutes of ischemia, reperfusion was induced by cutting the sutures and confirmed with resolution of ST segment elevation. Echocardiography was performed prior to and during ischemia and at 4 weeks after IR injury. Mice assigned to sham procedures received identical suture implantation and surgical manipulations 14 days later, but ischemia was not induced. Overall surgical mortality was less than 5%. All surgeries were performed by 2 surgeons who were blinded to animal genotypes. In a cohort of animals, cardioplegic solution was injected retrogradely through the aorta in situ at 24 hours after induction of ischemia, followed by sectioning of the left ventricle into slices, which were incubated in triphenyl tetrazolium chloride (TTC) for 30 minutes at 37°C. For infarct size calculation, TTC-stained slices were imaged and infarct area quantified as non–TTC-stained area as a ratio of total myocardial area, as previously described (60).

### Echocardiography.

2D-directed M-mode echocardiography was performed using a Vevo 2100 Imaging System (VisualSonics) equipped with a 30 MHz linear-array transducer, as previously described (59, 61). For echocardiographic studies on unstressed mice, mice were anesthetized with 100 mg/kg intraperitoneally injected tribromoethanol. For mice undergoing IR injury, echocardiography before and during ischemia was performed under isoflurane anesthesia (1.5%), and echocardiography at 4 weeks after IR injury was performed under 100 mg/kg intraperitoneally injected tribromoethanol anesthesia. The echocardiographer was blinded to animal genotype during image acquisition. Area-at-risk, left ventricular dimensions, wall thickness, heart rate, fractional shortening, EF, and volume calculations were performed by a blinded echocardiographer using the VevoStrain software (Visual Sonics), as described (59).

### Histology.

Histologic assessment with H&E staining and assessment of myocardial fibrosis with Masson’s trichrome staining and transmission electron microscopy were performed as previously described (18).

### Neonatal cardiac myocyte isolation.

Neonatal rat cardiac myocyte isolation was performed as previously described with the Worthington Neonatal Cardiomyocyte Isolation System (LK003300) (60). Hearts were harvested from 1-day-old neonatal rats and were subjected to trypsin digestion in a final concentration of 50 μg/ml in HBSS for 16–18 hours at 4°C after removal of the atria. Collagenase digestion (type II collagenase; 300 U/ml; Worthington) was conducted at 37°C for 45 minutes. Cardiomyocytes were seeded on collagen-coated 4-well chamber slides (Laboratory Tek) at a density of 10^5^ cells per cm^2^. On the second day, the culture medium was changed to the Rat Cardiomyocyte Culture Medium (Cell Applications Inc., R313-500) prior to immunofluorescence staining.

### Adenoviral studies.

Adenoviral vector encoding for CRYAB-R120G (18) was employed, as previously described.

### Biochemical fractionation into soluble and insoluble fractions.

We subjected cardiac tissues obtained from human hearts and from the remote left ventricular myocardium of mice subjected to IR injury (noninjured basal one-third) and cell extracts from neonatal rat cardiac myocytes to obtain soluble-insoluble fractions as previously described (18). Heart tissue was mechanically homogenized in homogenization buffer (0.3 M KCl, 0.1 M KH_2_PO_4_, 50 mM K_2_HPO_4_, 10 mM EDTA, 4 mM Na orthovanadate, 100 mM NaF, 1× protease inhibitor, pH to 6.5). Homogenized samples were passed through a mesh basket on ice, followed by collection of the lysate run-through which was incubated on ice for 30 minutes. An aliquot was transferred to another Eppendorf tube and 10% NP-40 was added for a final concentration of 1% NP-40. Samples were then incubated on ice for 30 minutes and spun at 16,000*g* for 15 minutes at 4°C. Supernatant was collected as soluble fraction. The pellet was washed 3 times with cold PBS (following addition of 1 ml PBS to each pellet and spin down at 16,000*g* for 10 minutes) followed by resuspension in 1% SDS, 10 mM Tris buffer to generate the insoluble fraction.

### Co-IP.

Frozen hearts were placed in RIPA buffer (Cell Signaling Technology, 9806) containing Protease and Phosphatase Inhibitor (Thermo Fisher, 78442) and homogenized with a mechanical homogenizer. Tissue lysis was pelleted at 600*g* for 5 minutes. The pellet was dissolved with PBS containing 1% SDS and 5 mM EDTA and sonicated. The solution was centrifuged at 16,000*g* for 5 minutes, and the supernatant was diluted by 10-fold with PBS containing 5mM EDTA. Protein A/G Sepharose (Abcam, ab193262) was incubated with 5 μg antibody or IgG in PBS for 30 minutes at room temperature with rotation. The immunocomplex was added to the lysis solution after 15 minutes incubation with rotation at room temperature. The beads were washed with PBS 4 times. The proteins were eluted by boiling in SDS sample buffer and analyzed by Western blot. The antibodies used in this experiment were as follows: anti-desmin (desmin [D93F5] XP rabbit mAb, 5332) and anti-CRYAB (Abcam, ab13496).

### Immunofluorescence analysis.

We performed immunohistochemistry on cells and myocardial tissues as we have previously described (18). Primary cultures of neonatal rat cardiac myocytes were fixed in 100% cold methanol for 20 minutes, followed by blocking with 1% normal serum in PBS for 1 hour at room temperature. Primary antibodies used were as follows: anti-desmin (Santa Cruz Biotechnology Inc., SC-7559), anti-ubiquitin (Abcam, ab134953), anti-polyubiquitin (Sigma-Aldrich, 04-262), anti-actin (MilliporeSigma, A2066), anti–α-actinin (Abcam, ab9465), and anti–αB-crystallin polyclonal antibody (ENZO Life, ADI-SPA-223-F) with overnight incubation at 4°C. Paraffin-embedded heart sections (4 μm thick) were subjected to deparaffinization using xylene and serial 100%, 90%, 70% and 50% EtOH treatment followed by hydration using deionized water and heat-induced epitope retrieval in Diva Decloaker Solution (Biocare Medical, DV2004MX). This was followed by blocking using 1% BSA (Sigma-Aldrich, A9647-100G), 0.1% Tween-20 (Sigma-Aldrich, P2287-500ML) in PBS (Corning, 21-040-CM), and 5% donkey serum. The slides were incubated overnight with primary antibody. The next day, after serial washes, samples were stained with secondary antibody and mounted with fluorescent 4’,6-diamidino-2-phenylindole mounting medium (Vector Labs, H-1200). For A11-staining (ThermoFisher, AHB005), the protocol was revised according to the vendors’ instructions. The antigen -retrieval solution was 0.1M glycine/PBS, at pH 3.5. Anti-oligomer A-11 antibody was used at 1–5 μg/mL concentration in 1:1mixture of blocking solution (1% BSA, 0.1% Tween-20 in PBS) and PBS. Confocal imaging was performed on a Zeiss confocal LSM-700 laser-scanning confocal microscope using 40×/1.3 oil immersion objectives, and images were processed using the Zen black software, version 3.05R.

### Striation and aggregate scoring.

To quantitate the mislocalization and aggregation of CRYAB client proteins desmin, actin, and α-actinin, we utilized the following scoring system: (a) For striation scoring, normal localization of proteins got scored as 0, and abnormal striation or mislocalization of proteins was scored as of 1. (b) For scoring aggregates, absence of aggregates was scored as 0 and presence of aggregates was scored as 2. Then, all the individual scores of the cardiomyocytes in the total field of view were added and divided by total number of cardiomyocytes. At least 3 images were assessed per sample. Image acquisition and quantitation were done by different operators where scoring was done blindly.

### Immunoblotting.

Immunoblotting was performed as previously described (18) with antibodies listed below. Specific antibodies employed are as follows: anti-SQSTM1/p62 antibody (Abcam, ab56416); αB-crystallin (CRYAB) polyclonal antibody (Enzo Life Sciences, ADI-SPA-223-F); anti-αB crystallin (pS59) antibody (Abcam, ab5577) (62); anti-αB crystallin (pS45) antibody (Abcam, ab5598) (62); anti-ubiquitin (Abcam, ab134953); anti–poly-ubiquitinylated proteins antibody, clone FK1 (Sigma-Aldrich, 04-262); anti-GAPDH antibody (Abcam, ab22555); anti-actin antibody (Sigma, A2066); anti-GFP antibody (Abcam, ab290), and anti-desmin antibody (Santa Cruz Biotechnology, SC-7559).

### Generation of crystallin constructs.

pHR-mCh-Cry2WT (Addgene 10221) and pHR-FUSN-mCh-Cry2WT (Addgene 10223) as generated by Clifford Brangwynne (Department of Chemical and Biological Engineering, Princeton University, Princeton, New Jersey, USA) (39), were obtained and verified by sequencing. For phase-separation assay in the live cells, we generated a pHR-CRYAB-mCh-Cry2 construct using the In-Fusion Snap Assembly Kit (Takara, 638945). CRYAB S59A, S59D, and R120G+S59A mutations were introduced in pHR-CRYAB-mCh-Cry2 construct using the QuikChange II Site-Directed Mutagenesis Kit (Agilent, 200523). These constructs were subsequently cloned into pcDNA 3.1 mammalian vector for the expression in HEK293A cells. Constructs coding for eGFP-CRYAB, eGFP-CRYAB S59A, eGFP-CRYAB S59D, eGFP-CRYAB-R120G+S59A and eGFP-CRYAB-R120G+S19A+S45A+S59A were generated as described above in a pcDNA3.1 backbone for protein aggregation studies and cell death assessment.

### Assessment of protein aggregation and cell death.

HEK293 cells were transfected with various GFP-tagged CRYAB constructs using Lipofectamine 3000 reagent (Thermo Fisher, L3000) and cells imaged for GFP at 24 hours after transfection under Zeiss 700 confocal microscope for the presence of protein aggregation. Cells were harvested at 48 hours for cell-death assay. Cell death was assessed using a fluorometric assay with LIVE/DEAD Viability/Cytotoxicity Kit for mammalian cells (Thermo Fisher, L3224), as we have previously described (18).

### Studies with optoDroplet constructs.

CRYAB constructs were transfected into HEK 293A cells using Effectene Transfection Reagent (QIAGEN, 301425) and live-cell imaging was performed using 35 mm glass-bottom dishes at 24 hours after transfection using 40× oil immersion objective of the Nikon A1R Confocal Imaging System (equipped with 37°C stage) at the Center for Cellular Imaging (WUCCI) at Washington University in St. Louis School of Medicine. Cells were imaged with 2 laser wavelengths (488 nm for Cry2 activation and 561 nm for mCherry imaging) with laser power of 10% for the 488 nm. Various parameters were quantified using ImageJ (NIH) by adjusting the threshold to ensure uniform cut-off values at the *t* = 0 and *t* = 300 seconds time-point images. Average condensate area was determined by total area of the fluorescence signal divided by number of condensates before or after 300 seconds of the blue light activation. Average number of condensates per cell was obtained by dividing total number of condensates by the number of cells before or after 300 seconds of the blue light activation.

### Studies with FRAP.

FRAP was performed on OptoIDR constructs as described above while imaging with 100× objective lens of the Nikon A1R Confocal Imaging System, using a 488 nm laser at 50% intensity and 561 nm laser at 50% intensity for 1 minute, selecting a region of interest of approximately 1 μm in diameter; and fluorescence recovery was monitored. Intensity traces were collected using ImageJ and normalized to prebleaching intensity (set at 100%). Only condensates with reduction in intensity to less than 10% (as compared with prebleach) after photobleaching were selected for further analysis.

### Studies with 25-HC.

25-HC (H1015-100MG, Sigma-Aldrich) was dissolved in 100% ethanol (final concentration 20 mg/mL) to make a stock solution. For in vivo experiments, the stock solution was diluted in sterile PBS for injection. A dose of 10 mg/kg or equivalent vehicle control was administered every other day via intraperitoneal injection beginning at day 4 after closed-chest IR injury for 3.5 weeks. Mice were weighed prior to injection and food intake was monitored through the experiment.

### Statistics.

Data are presented as mean ± SEM. All measurements were obtained from separate biological replicates. Statistical analyses were performed using GraphPad Prism, version 9. Data were tested for assumptions of normality with the Shapiro-Wilk normality test. Statistical significance was assessed with unpaired 2-tailed Student’s *t* test for comparison between 2 groups or 1-way or 2-way ANOVA for assessing differences across multiple groups followed by post hoc testing. A nonparametric test was performed if data were not normally distributed. A 2-tailed *P* value of less than 0.05 was considered statistically significant.

### Study approval.

All animal studies were approved by the IACUC at Washington University School of Medicine. Studies on human tissue were performed on deidentified human samples, and these studies were deemed exempt by the IRB at Washington University School of Medicine.

### Data availability.

Values for all data points in graphs are reported in the Supporting Data Values file. Data are available upon request.

## Author contributions

MI, DRR, XM, XG, WN, CZ, LF, JTM, CJW, HN, Aaradhya Diwan, RPC, and MK performed experiments and acquired and analyzed the data. AK and JN acquired and analyzed the data. BR provided critical reagents, interpreted the data, and revised the manuscript. MEY, MA, SS, KBM, DFC, and AJ interpreted the data and revised the manuscript. KM and Abhinav Diwan conceived the experiments, analyzed the data, and drafted the manuscript. KM and Abhinav Diwan supervised the work. MI and DRR are co–first authors with order assigned as such based on MI having initiated the project.

## Supplementary Material

Supplemental data

Unedited blot and gel images

Supplemental video 1

Supplemental video 2

Supplemental video 3

Supplemental video 4

Supplemental video 5

Supplemental video 6

Supplemental video 7

Supplemental video 8

Supplemental video 9

Supplemental video 10

Supplemental video 11

Supplemental video 12

Supplemental video 13

Supplemental video 15

Supporting data values

## Version 1 02/11/2025

In-Press Preview

## Version 2 04/01/2025

Electronic publication
