# Supplementary material for: Phosphorylation of CRYAB induces a condensatopathy to worsen post–myocardial infarction left ventricular remodeling
Source: J Clin Invest. 2025 Feb 11;135(7):e163730. doi: 10.1172/JCI163730 (PMC11957698; doi:10.1172/JCI163730)
Supplement: Supplemental data [file jci-135-163730-s056.pdf]

Table of Contents for Supplementary Data

Supplementary Fig. S1: Desmin localizes to protein-aggregates and pre-amyloid oligomers are observed in human ischemic cardiomyopathy…………………………………… Page 2

Supplementary Fig. S2: CRYAB phosphorylated at serine-59 partitions to the insoluble fraction in the myocardium of young adult C57BL6J WT mouse subjected to closed chest ischemia-reperfusion (IR) with development of ischemic cardiomyopathy…………Pages 3, 4

Supplementary Fig. S3: R120G protein is prominently phosphorylated at S59. …… Page 5

Supplementary Fig. S4: Preventing phosphorylation at all three serine residues in CRYAB (S19, S45 and S59) reduces cell death in CRYAB R120G………………………………Page 6

Supplementary Fig. S5: N-terminus, ACD, and C-terminus domains of CRYAB undergo phase separation……………………………………………………………………… Page 7

Supplementary Fig. S6: Serine to alanine change at position 59 prevent phosphorylation at this residue in CRYAB……………………………………………………………….. Page 8

Supplementary Fig. S7: Crispr-Cas9 knock-in of phosphorylation-deficient serine to alanine change at position 59 (S59A) and phospho-mimetic serine to aspartic acid change (S59D) does not alter total CRYAB and desmin abundance……………………………….. Page 9

Supplementary Fig. S8. Serine-to-aspartic acid mutation at CRYAB residue 59 results in increased polyubiquitinated proteins in the mouse heart……………………….. Pages 11, 12

Supplemental Figure S9. Phosphorylation-deficient serine-to-alanine mutation at CRYAB residue 59 (S59A) does not alter initial infarct size in mice undergoing IR injury…. Page 13

Supplementary Fig. S10: 25-HC does not affect phase separation or dynamicity of CRYAB S59D………………………………………………………………………………………. Page 14

Supplementary Fig. S11: 25-HC treatment reduces overall pS59CRYAB in the WT C57BL6J mice subjected to IR injury…………………………………………………………… Page 15

Supplemental Figure S12. 25-hydroxycholesterol treatment reduces phosphorylation of the serine residue at position 59 of CRYAB in sham-operated mice …………………… Page 16

Table S1: Characteristics of individuals whose human heart samples were included in the study…………………………………………………………………………………….. Page 17

Table S2: Morphometric and M-mode echocardiographic data for CRYAB WT, S59A homozygous knock-in, and S59D homozygous knock-in mice at 10 weeks of age….. Page 18

Table S3: Morphometric and echocardiographic data for CRYAB WT, S59A homozygous knock-in, and S59D homozygous knock-in mice subjected to IR injury……………… Page 19

Table S4: Morphometric and echocardiographic data for C57 WT mice that underwent IR-surgery and treated with diluent or 25-HC…………………………………………….. Page 20

Supplementary Videos S1-S14.………………… Available as Separate files

Supplementary Figure S1

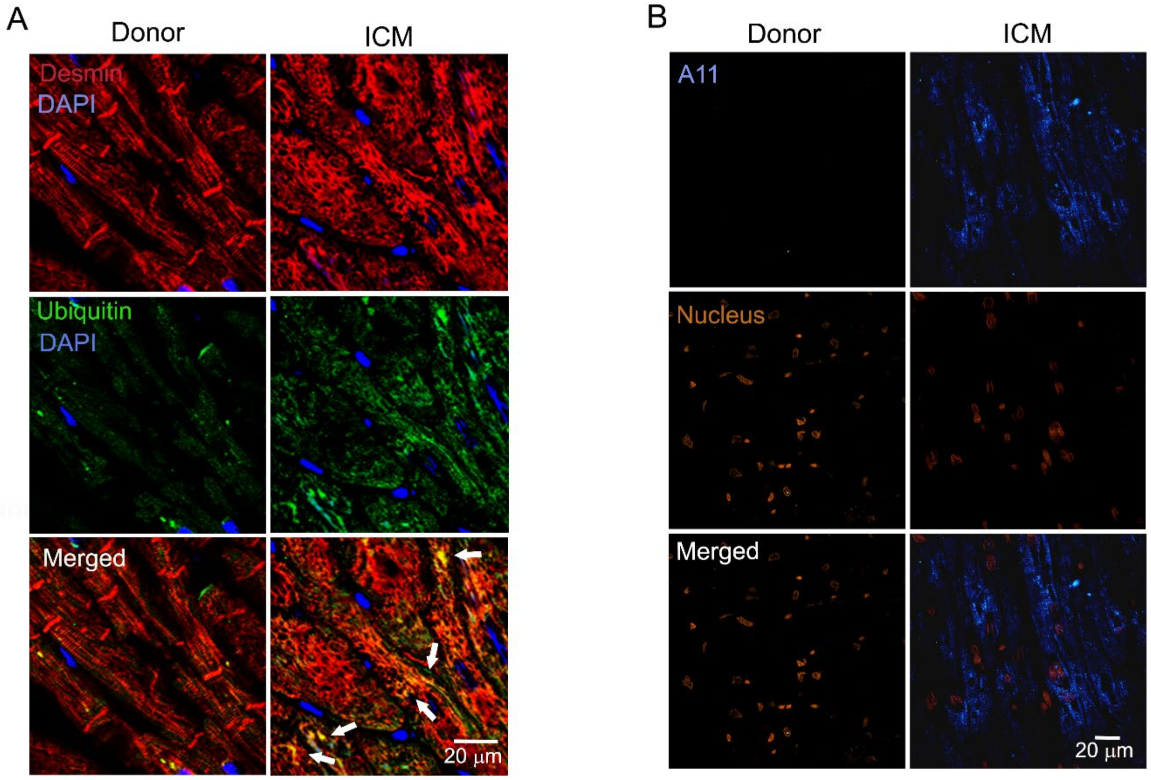

Supplementary Fig. S1: Desmin localizes to protein-aggregates and pre-amyloid oligomers are observed in human ischemic cardiomyopathy. A. Representative immunohistochemical images from left ventricular myocardium of individuals evaluated as controls (donor) or patients with end-stage ischemic cardiomyopathy (ICM) stained for desmin and polyUb (ubiquitin). Arrows point to desmin, which is mis-localized from its physiologic location on Z-discs and intercalated discs in donor myocardium to protein-aggregates stained with polyUb antibody in ICM myocardium. DAPI stains nuclei. Representative of n=3 hearts/group. **B.** Immunohistochemical staining for anti-oligomer A11 antibody in donor and ICM heart samples, demonstrating presence of pre-amyloid oligomers structures in ICM samples (pseudo-colored blue, arrows). DAPI stained nuclei are pseudo-colored orange. Representative of n=5 hearts/group.

Supplementary Figure S2

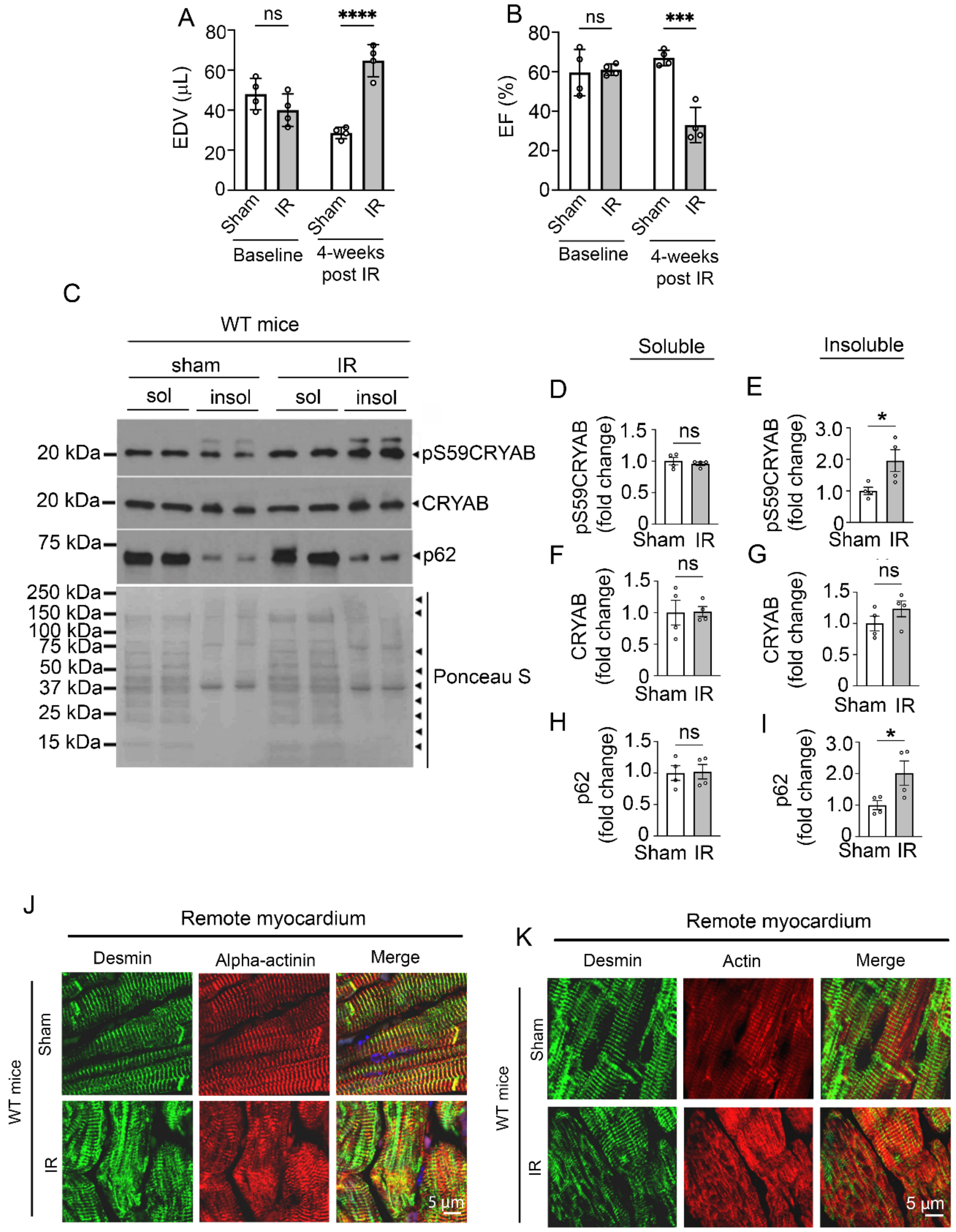

Supplementary Fig. S2: CRYAB phosphorylated at serine-59 partitions to the insoluble fraction in the myocardium of young adult C57BL6J WT mouse subjected to closed chest ischemia-reperfusion (IR) with development of ischemic cardiomyopathy. A, B) Left ventricular end-diastolic volume (EDV) (A) and left ventricular ejection fraction (EF, %) (B) at

baseline and at 4 weeks after closed chest IR (90 minutes of ischemia followed by reperfusion) in male C57BL6J WT young adult mice that were subjected to IR injury or sham procedure. *** indicates P value < 0.001 and **** indicates P value <0.0001 by t-test. **C-I)** Representative (C) immunoblot and quantitation depicting the abundance of pS59CRYAB (D, E), CRYAB (F, G) and p62 (H, I) in the soluble (*left* panels) and insoluble (*right* panels) biochemical fractions from the remote myocardium of the C57BL6J WT young adult mice collected 4 weeks after IR or sham procedure as in A, B. Ponceau S is shown as loading control. * indicates P<0.05 by t-test. **J, K)** Representative images demonstrating expression of desmin and α-actinin (J), and of desmin and actin (K) in the myocardium from C57BL6J mice 4 weeks after being subjected to IR injury. DAPI stains nuclei.

Supplementary Figure S3

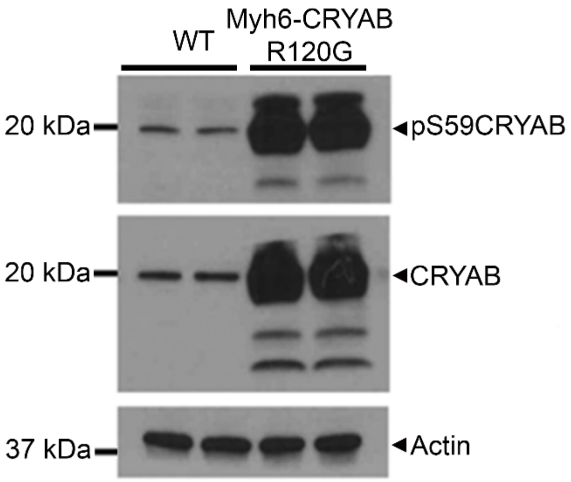

Supplementary Fig. S3: R120G protein is prominently phosphorylated at S59. **A)** Representative image showing expression of CRYAB and pS59CRYAB in crude extracts from 40-week-old Myh6-CRYABR120G mouse hearts or from C57BL6J WT mouse hearts as control. Actin is shown as the loading control.

Supplemental figure S4

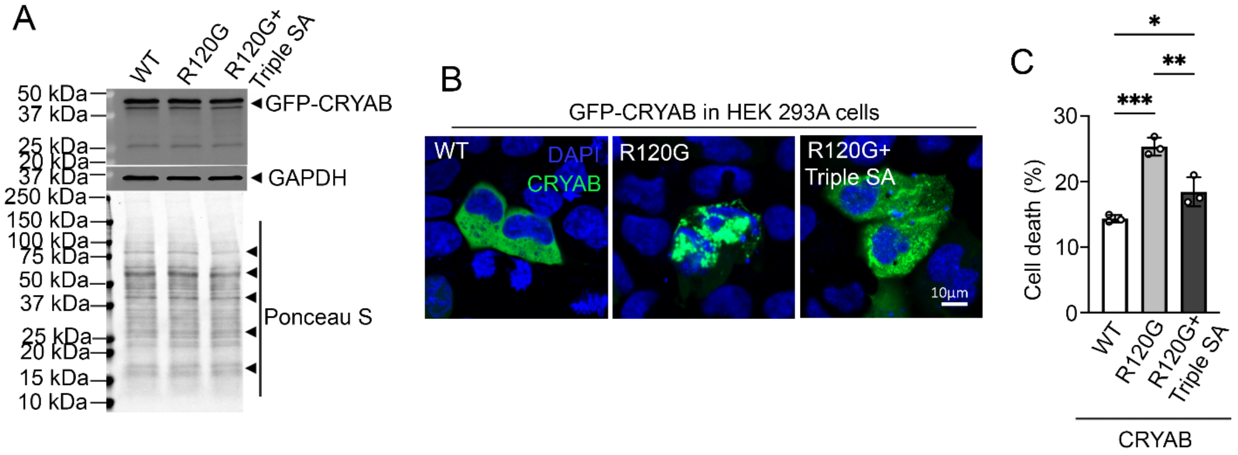

Supplementary Fig. S4: Preventing phosphorylation at all three serine residues in CRYAB (S19, S45 and S59) reduces cell death in CRYAB R120G. A) Immunoblot (A) demonstrating expression of GFP-fusion proteins in HEK293A cells transfected with GFP-tagged wild-type CRYAB, R120G mutant or the R120G and S19A, S45A, S59A triple mutant proteins (Triple SA). **B, C)** Representative immunofluorescence images (B) for detection of protein-aggregates with quantitation (C) of % cell death. * Indicates $p<0.05$, ** indicates $p<0.01$, and *** indicates $P<0.001$ by Tukey's post-hoc test after one-way ANOVA. Nuclei are blue (DAPI).

Supplementary Figure S5

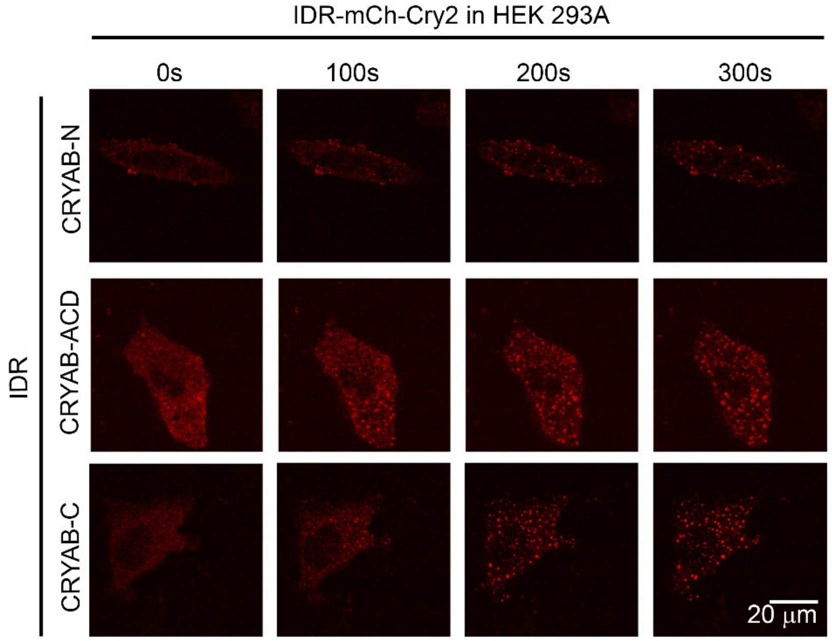

Supplementary Fig. S5: N-terminus, ACD, and C-terminus domains of CRYAB undergo phase separation. Representative time lapse images at t=0s, 100s, 200s, and 300s after light activation in HEK293A cells transfected with constructs generated with CRYAB N-terminus, Alpha-crystallin domain (ACD), and C-terminus domains as the 'IDR' in the optoIDR constructs.

Supplementary Figure S6

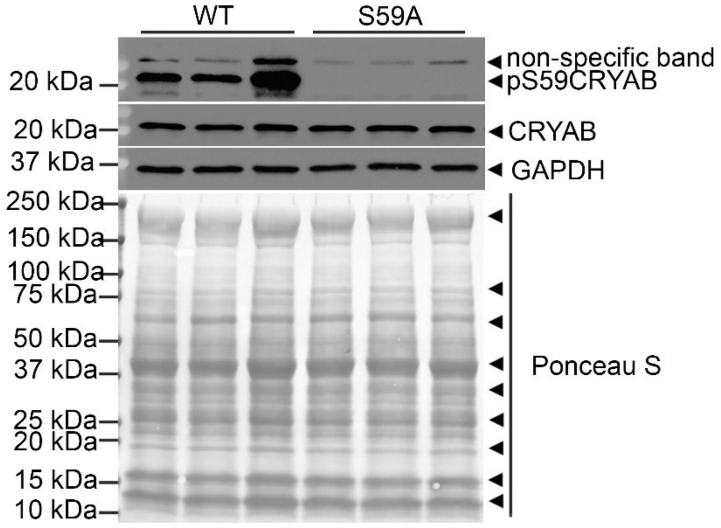

Supplementary Fig. S6: Serine to alanine change at position 59 prevents phosphorylation at this residue in CRYAB. Representative immunoblot demonstrating loss of immunodetectable band with an antibody that recognizes CRYAB phosphorylated on serine 59 (pS59-CRYAB), total CRYAB and GAPDH in myocardial extracts from mice homozygous for alleles bearing knock-in of alanine residue at this position. The specific band indicating pS59-CRYAB is indicated. Ponceau S is shown as loading control.

Supplementary Figure S7

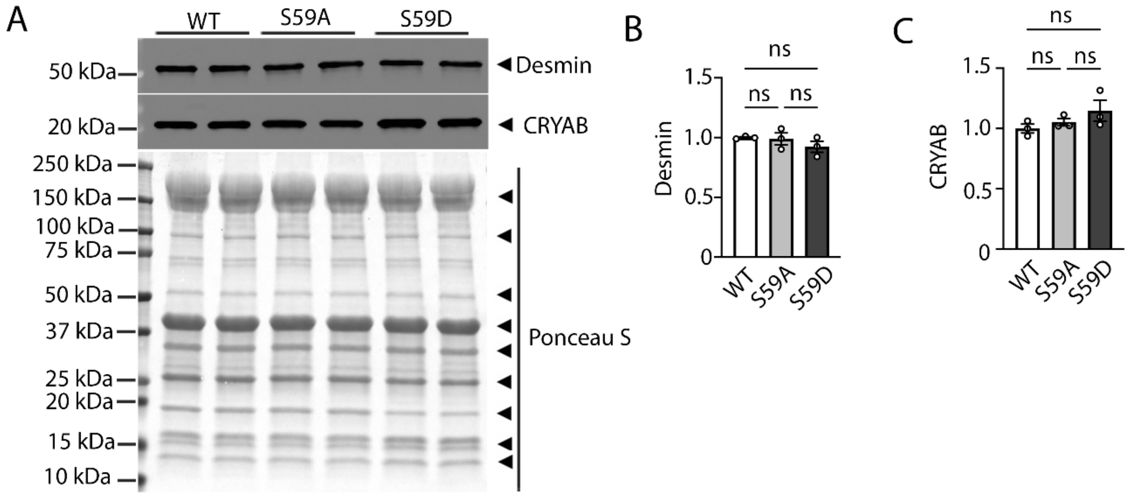

Supplementary Fig. S7: Crispr-Cas9 knock-in of phosphorylation-deficient serine to alanine change at position 59 (S59A) and phospho-mimetic serine to aspartic acid change (S59D) does not alter total CRYAB and desmin abundance. A-C) Representative immunoblot demonstrating expression of CRYAB and desmin in myocardial extracts from young adult mice homozygous for S59A or S59D CRYAB alleles or bearing wild-type CRYAB (WT) with quantitation of desmin (B) and CRYAB abundance (C) both expressed as fold over WT. 'ns' indicates no statistically significant differences were noted by Tukey's post-hoc test after one-way ANOVA analyses. Ponceau S is shown as loading control.

Supplementary Figure S8

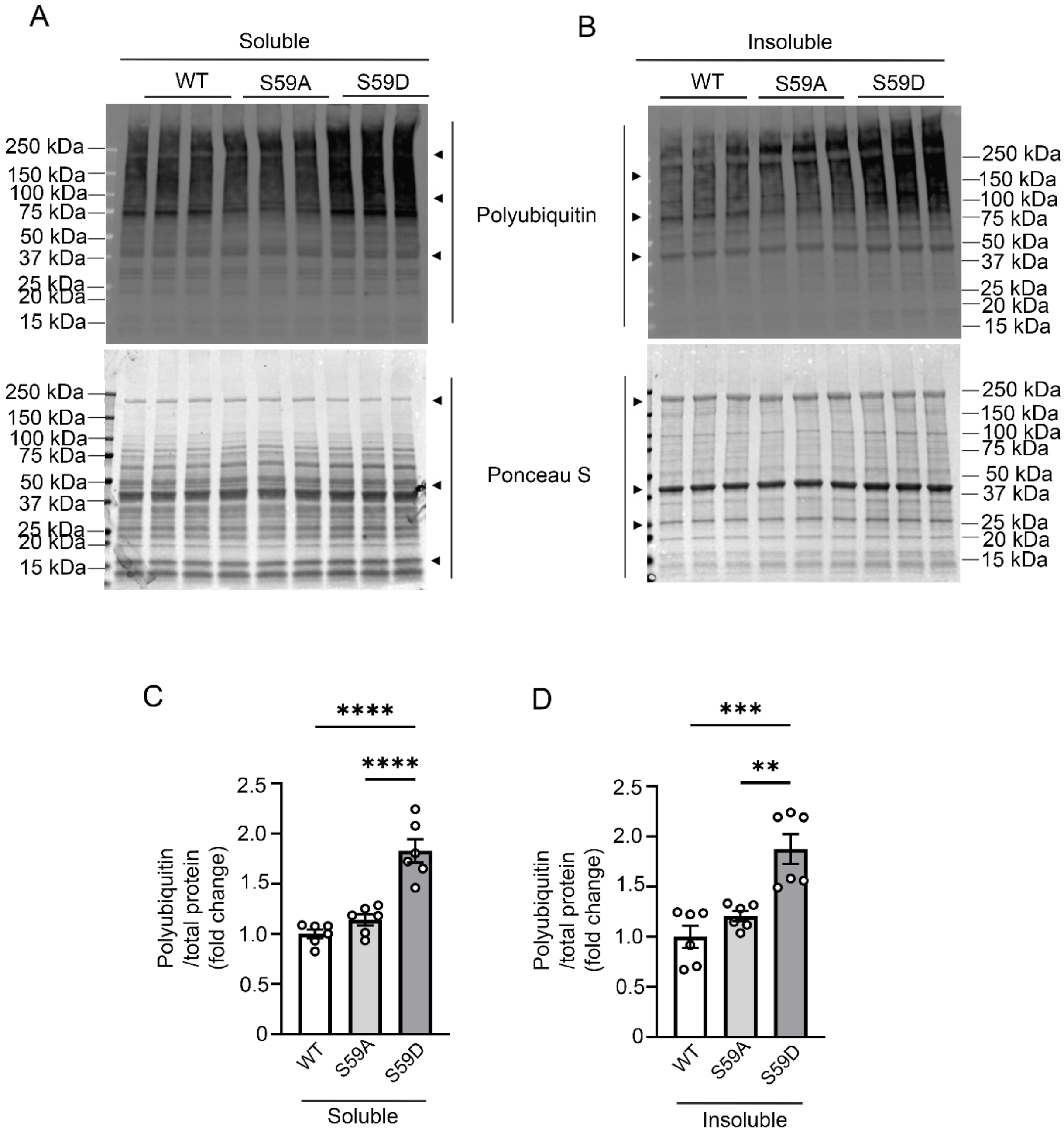

Supplementary Fig. S8. Serine-to-aspartic acid mutation at CRYAB residue 59 results in increased polyubiquitinated proteins in the mouse heart. A, B) Representation immunoblots depicting levels of poly-ubiquitinated proteins from the mouse heart in the NP-40 soluble (A) and NP-40 insoluble (B) fractions from wild-type mice (WT), mice homozygous for a phosphorylation deficient serine to alanine mutation at CRYAB position 59 (S59A) and mice homozygous for a phospho-mimetic serine to aspartic acid mutation at CRYAB position 59 (S59D). Ponceau S staining is shown as a loading control. **C, D)** Quantitation of poly-ubiquitinated proteins from the NP-40 soluble (C) and NP-40 insoluble (D) fractions from WT,

S59A, and S59D hearts. Poly-ubiquitinated levels are normalized to total protein levels as assessed by Ponceau S staining. ** indicates $P<0.01$, **** indicates $P<0.0001$ by Tukey's post-hoc test after one-way ANOVA.

Supplementary Figure S9

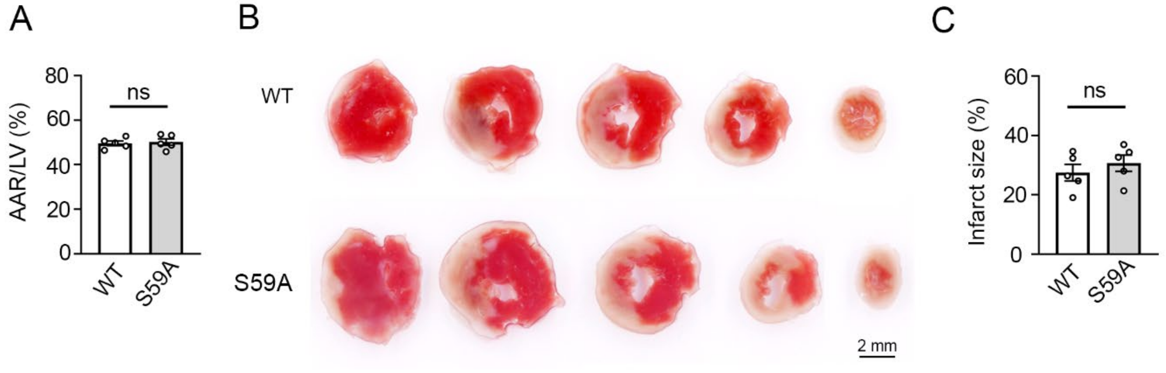

Supplemental Figure S9. Phosphorylation-deficient serine-to-alanine mutation at CRYAB residue 59 (S59A) does not alter initial infarct size in mice following IR injury. Wild-type (WT) mice and mice homozygous for S59A mutation (S59A) underwent suture implantation followed by 90 minute closed-chest ischemia-reperfusion (IR) injury 2 weeks later, as shown in Figure 5A. Mice were sacrificed 24 hours after closed-chest IR injury, and hearts underwent triphenyl tetrazolium chloride (TTC) staining to assess infarct area. **A)** Quantitative assessment of area-at risk (AAR) by echocardiography with LAD occlusion during closed-chest IR injury. **B)** Representative images of TTC staining from hearts harvested 24 hours after closed-chest IR injury from mice homozygous for S59A mutation (S59A) or wild-type (WT) control mice. **C)** Quantitation of infarction size at 24 hours post-IR injury by TTC staining in B in either S59A mouse hearts or WT control hearts. ns indicates P>0.05 by t-test.

Supplemental Figure S10

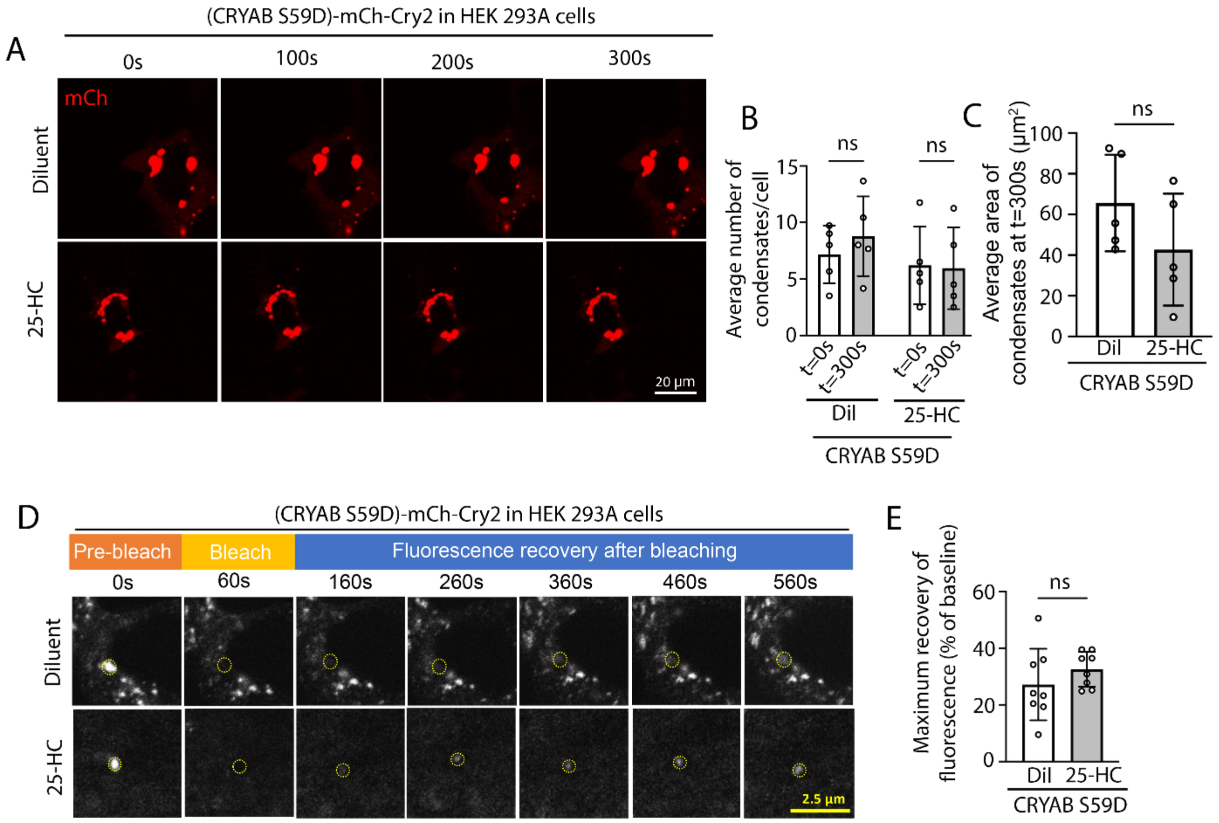

Supplementary Fig. S10: 25-HC does not affect phase separation or dynamicity of CRYAB S59D. A) Representative time-lapse images at t=0s, 100s, 200s, and 300s after light activation in HEK293A cells transfected with constructs generated with CRYAB S59D, the phospho-mimetic mutant as the ‘IDR’ in the optoIDR constructs. **B**) Average number of condensates/cell at t=0 vs. t=300s in cells treated as in E. ‘ns’ indicates not significant by t-test. **C)** Average area of condensates/cell at t=300s in cells treated in A. ‘ns’ indicates not significant by t-test. **D)** Representative images demonstrating recovery of fluorescence after photobleaching in HEK 293A cells transfected with mCherry-Cry2 fused optoIDR constructs generated with CRYAB S59D, the phospho-mimetic mutant. Representative images demonstrate area of photobleaching (marked with a dotted circle) prior to (pre-bleach), immediately after, and at 100, 200, 300, 400 and 500 seconds (s) after photobleaching was terminated. Intensity at various time points is depicted as a fraction of intensity prior to bleaching (set at 100%). **E)** Quantitation of fluorescence recovery (maximum minus immediately post-bleach) in CRYAB S59D variants indicated in D. ‘ns’ indicates not significant by t-test.

Supplementary Figure S11

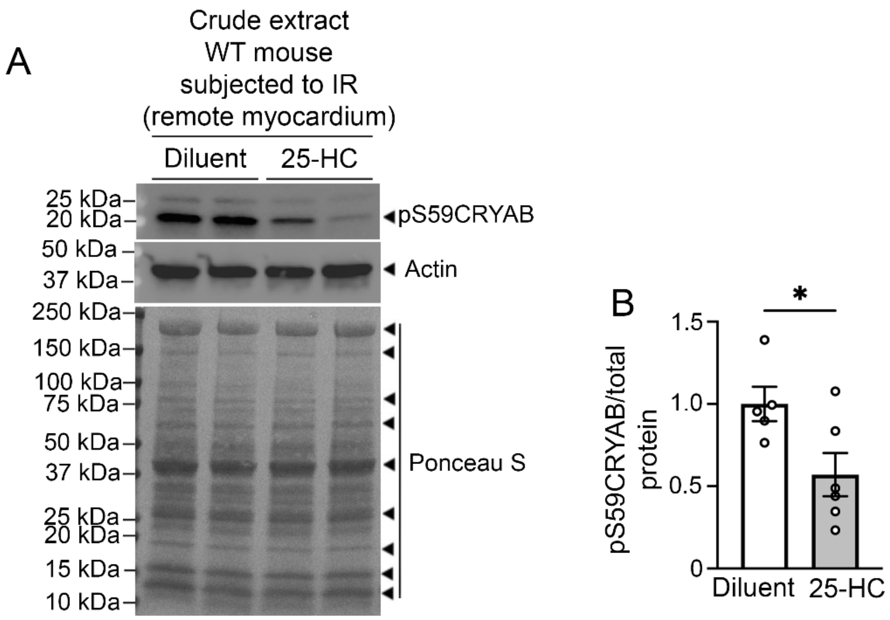

Supplementary Fig. S11: 25-HC treatment reduces overall pS59CRYAB in the WT C57BL6J mice subjected to IR injury. A) Representative immunoblot (A) and quantitation (B) of pS59CRYAB in crude extracts of remote myocardium from WT C57BL6J young adult male mice that were treated with 25-HC or diluent after IR injury as shown in Figure 8A. * indicates $p<0.05$ by t-test. Actin and Ponceau S were used as loading controls.

Supplementary Figure S12

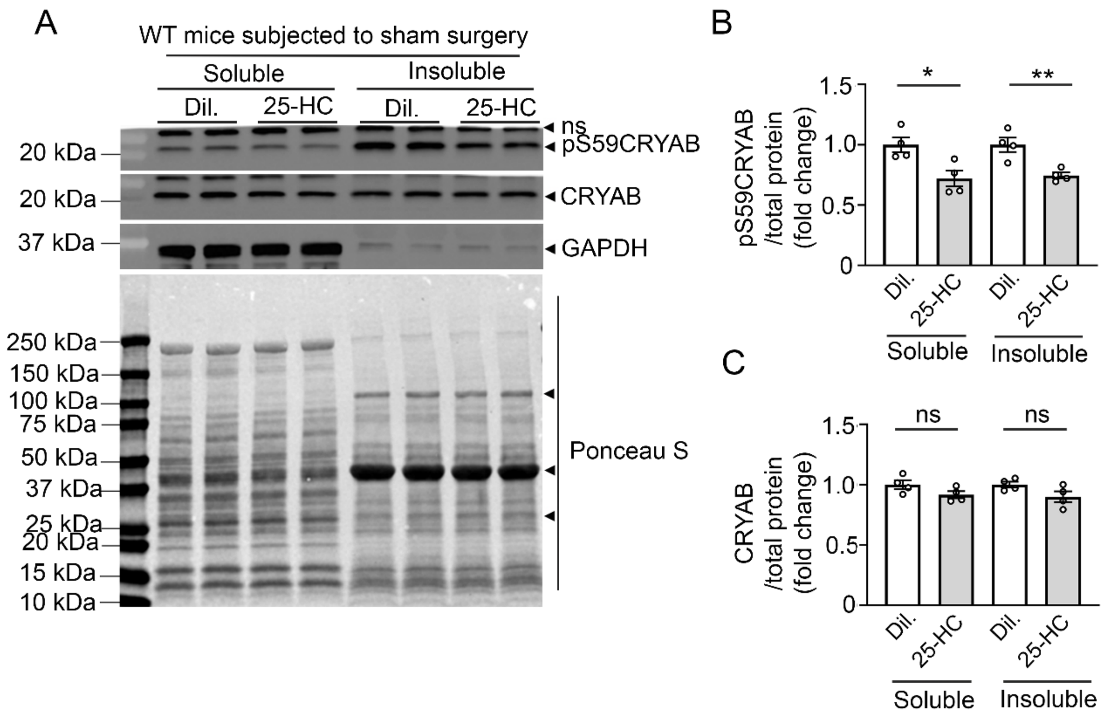

Supplemental Figure S12. 25-hydroxycholesterol treatment reduces phosphorylation of the serine residue at position 59 of CRYAB in sham-operated mice. A) Representation immunoblots showing the levels of CRYAB phosphorylated at serine residue 59 (pS59CRYAB), total CRYAB expression (CRYAB), and GAPDH from the NP-40 soluble and NP-40 insoluble fractions isolated from the hearts of wild type mice which underwent a sham closed-chest ischemia-reperfusion procedure followed by every-other-day 25-hydroxycholesterol (25-HC) treatment from post-operative day 4 to day 28 (as in Figure 8), or diluent control treatment for the same period (Dil). Ponceau S staining is shown as a loading control. **B, C)** Quantitation of pS59CRYAB level (B) or total CRYAB level (C) from the soluble and insoluble fractions from hearts of mice under indicated 25-HC or diluent treatments. CRYAB and pS59CRYAB levels are normalized to total protein levels as measure by Ponceau S staining. Protein levels are shown as a fold change relative to diluent control expression. ns indicates $P>0.05$, * indicates $P<0.05$, ** indicates $P<0.01$ by t-test.

Supplementary tables:

Table S1: Characteristics of individuals whose human heart samples were included in the study.

**Individual Characteristics**	**Ischemic Cardiomyopathy (n=8)**	**Non-failing Donor (n=8)**	**Statistical Comparison**
Male Gender/Total Sample (N)	5/8	6/8	NS
Age (years)	57 ± 3	52 ± 2	NS
BMI	27.79 ± 1.02	29.27± 3.74	NS
LV Mass Index (g/kg)	175.97 ± 3.76	105.38 ± 1.86	P<0.0001
LVEDD (cm)	6.32 ± 0.28	4.03 ± 0.36	P<.01
LVEF (%)	22.53 ± 3.85	60.13 ± 2.61	P<0.0001
History of Diabetes	2/8	2/8	NS
History of HTN	4/8	1/8	NS
History of ACE-I/ARB use	4/8	0/8	NS
History of Beta-blocker use	4/8	0/8	NS

All data for continuous variables are mean±SEM. P values reported are by two-tailed t-test for continuous variables and by Fisher's exact test for categorical variables. NS indicates not significant.

Table S2: Morphometric and M-mode echocardiographic data for CRYAB WT, S59A homozygous knock-in, and S59D homozygous knock-in mice at 10 weeks of age.

	CRYAB WT (n=6)	CRYAB S59A (n=8)	CRYAB S59D (n=8)
**BW (g)**	22.95 ± 1.61	22.28 ± 1.06	21.09 ± 1.39
**HW/BW (mg/g)**	4.53 ± 0.11	4.71 ± 0.16	4.78 ± 0.07
**HW/TL (mg/mm)**	6.50 ± 0.33	6.29 ± 0.18	6.36 ± 0.39
**LVIDd (mm)**	3.29 ± 0.09	3.13 ± 0.06	3.20 ± 0.10
**LVIDs (mm)**	1.76 ± 0.10	1.62 ± 0.04	1.75 ± 0.09
**FS (%)**	46.65 ± 1.81	48.43 ± 0.79	45.69 ± 1.19
**LV mass (mg)**	63.41 ± 2.98	57.37 ± 1.55	63.34 ± 4.41
**HR (beats per min)**	625± 14	593 ± 8	608 ± 11

Data represent mean ± SEM. No significant differences were found between groups in ordinary one-way ANOVA. BW= Body Weight, HW= Heart Weight, TL= Tibia Length, LVIDd= Left Ventricular Internal Diameter End Diastole, LVIDs= Left Ventricular Internal Diameter End Systole, FS= Fractional Shortening, LV mass= Left-Ventricular mass based on M-mode echocardiography measurements, HR= Heart rate.

Table S3: Morphometric and echocardiographic data for CRYAB WT, S59A homozygous knock-in, and S59D homozygous knock-in mice subjected to ischemia-reperfusion injury.

	CRYAB WT (n=19)	CRYAB S59A (n=10)	CRYAB S59D (n=9)
**BW (gm)**	23.20 ±0.68	22.43 ± 0.78	25.64 ± 0.96
**HW/ BW (mg/gm)**	5.30 ± 0.10	5.30 ± 0.08	5.36 ± 0.14
**HW/ TL (mg/mm)**	7.18 ± 0.13	6.85 ± 0.24	7.81 ± 0.22*
**Lung W/TL (mg/mm)**	7.74 ± 0.18	7.09 ± 0.16 ǂ	7.72 ± 0.14
**Liver W/TL (mg/mm)**	61.70 ± 2.18	58.18 ± 2.80	62.34 ± 3.69
**LVEDV pre-isch. (µL)**	39.80 ± 1.45	40.83 ± 2.50	47.50 ± 3.86
**LVEDV 4W post-isch. (µL)**	52.28 ± 2.10	48.61 ± 2.94	60.35 ± 3.70*
**LVESV pre-isch. (µL)**	14.73 ± 0.90	15.60 ± 1.91	18.35 ± 2.70
**LVESV 4W post-isch. (µL)**	32.55 ± 2.00	26.90 ± 2.03	39.90 ± 3.43**
**LVEF pre-isch (%)**	62.74 ± 1.71	62.50 ± 2.40	62.35 ± 2.79
**LVEF 4W post-isch. (%)**	38.16 ± 1.49	44.91 ± 1.10ǂ	34.13 ± 1.85***
**LVM 4W post-isch. (mg)**	93.45 ± 2.03	89.91 ± 2.72	105.66 ± 3.71###***

Data represent mean ± SEM. All statistical comparisons were done with one-way ANOVA followed by post-hoc testing with Tukey's test. P<0.05 is represented by ǂ for WT vs S59A groups, # for WT vs S59D groups, and * for S59A vs S59D groups. P<0.01 is represented by ** for S59A vs S59D groups. P<0.001 is represented by ### for WT vs S59D groups, and *** for S59A vs S59D groups. BW= Body Weight, HW= Heart Weight, Lung W= Lung Weight, Liver W: Liver Weight, TL= Tibia Length, LVEDV= Left Ventricular End Diastolic Volume, 4W= 4-weeks. Pre-isch. = Pre-ischemia, post-isch. = Post-ischemia, LVESV= Left Ventricular End Systolic Volume, LVEF= Left Ventricular Ejection Fraction, FS= Fractional Shortening, LVM= Left-Ventricular mass based on echocardiography measurements.

Table S4: Morphometric and echocardiographic data for C57BL6J WT mice that underwent IR-surgery and treated with diluent or 25-HC.

	C57 WT, Diluent (n=8)	C57 WT, 25-HC (n=8)
**BW (gm)**	27.67 ± 0.44	26.27 ± 0.57
**HW/ BW (mg/gm)**	5.11 ± 0.19	4.32 ± 0.52
**HW/ TL (mg/mm)**	7.94 ± 0.42	7.13 ± 0.19
**Lung W/TL (mg/mm)**	7.72 ± 0.22	7.00 ± 0.14*
**Liver W/TL (mg/mm)**	58.07 ± 2.34	57.15 ± 1.50
**LVEDV pre-isch. (mL)**	44.75 ± 3.67	39.31 ± 1.99
**LVEDV 4W post-isch. (mL)**	86.16 ± 8.09	51.89 ± 4.09***
**LVEF pre-isch. (%)**	59.23 ± 1.50	62.40 ± 2.87
**LVEF 4W post-isch. (%)**	26.77 ± 1.22	43.70 ± 2.48****
**Area-at-risk/LV (%)**	37.93 ± 3.10	41.50 ± 11.81

Data represent mean ± SEM. * denotes $p<0.05$, *** denotes $p<0.001$, **** denotes $p<0.0001$ by t-test. BW= Body Weight, HW= Heart Weight, Lung W= Lung Weight, Liver W: Liver Weight, TL= Tibia Length, LVEDV= Left Ventricular End Diastolic Volume, 4W= 4-weeks. Pre-isch. = Pre-ischemia, post-isch. = Post-ischemia, LVEF= Left Ventricular Ejection Fraction based on echocardiography measurements.

Supplementary Videos S1-S14. Representative videos depicting light induced phase separation of indicated optoIDR constructs, along with positive control (FUS-N) and negative control (Cry2).
