# Supplementary material for: Phosphorylation of CRYAB induces a condensatopathy to worsen post–myocardial infarction left ventricular remodeling
Source: J Clin Invest. 2025 Feb 11;135(7):e163730. doi: 10.1172/JCI163730 (PMC11957698; doi:10.1172/JCI163730)

Uncropped gels western blots figure 1C

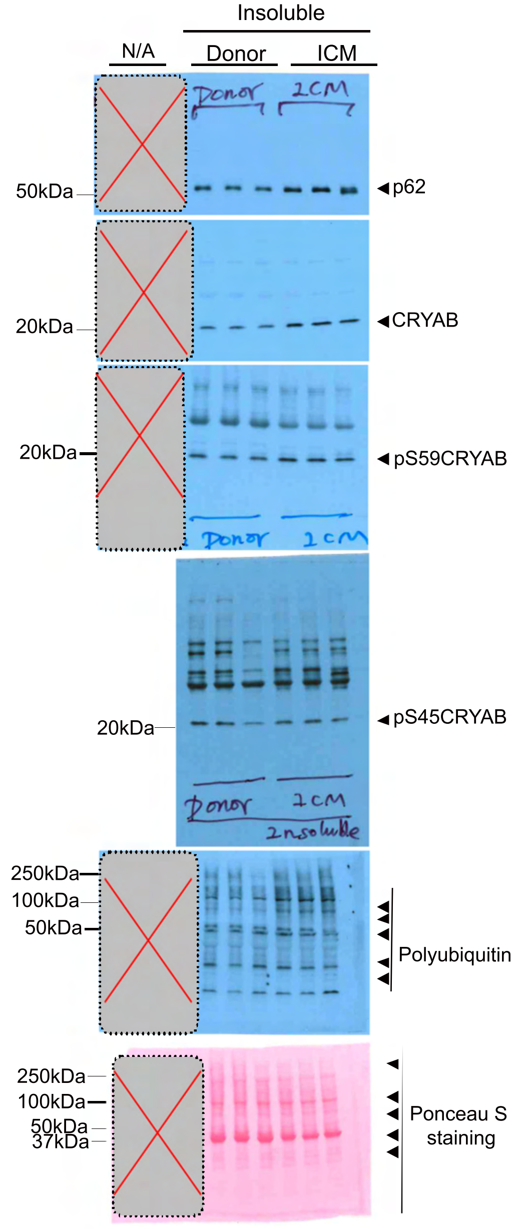

Uncropped gels western blots figure 1H

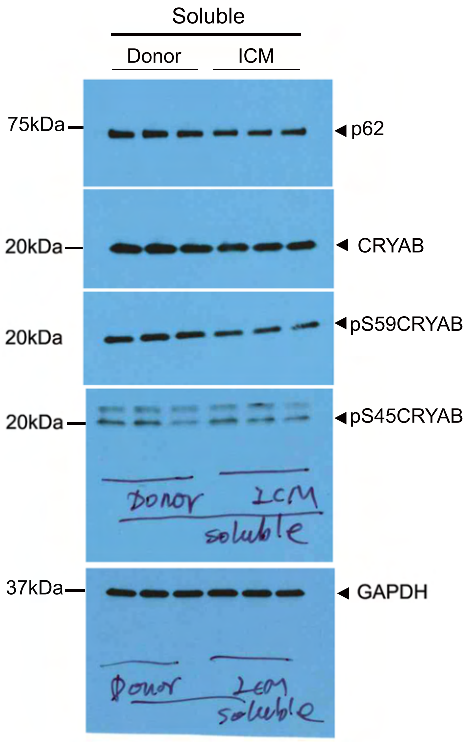

Note: N/A lanes are not applicable to this study and are marked with a cross

Uncropped gels western blots for figure 2A

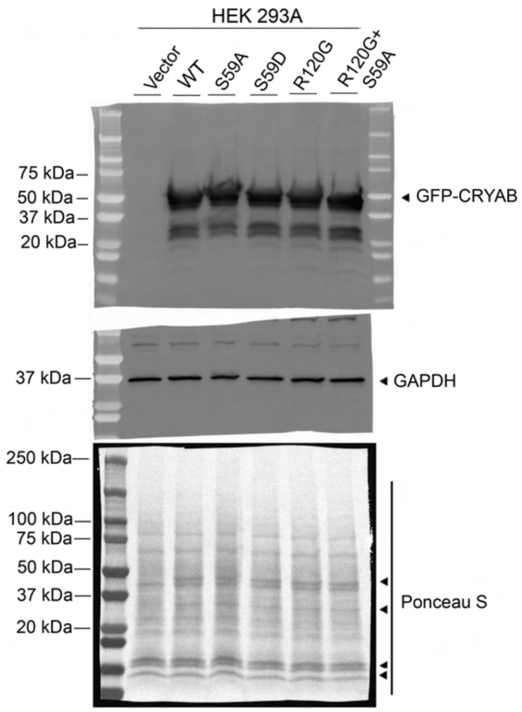

Uncropped gels western blots figure 4B

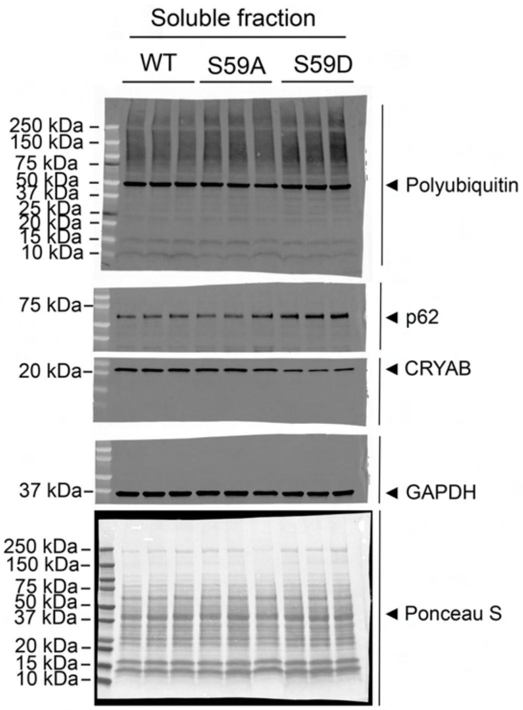

Uncropped gels western blots figure 4C

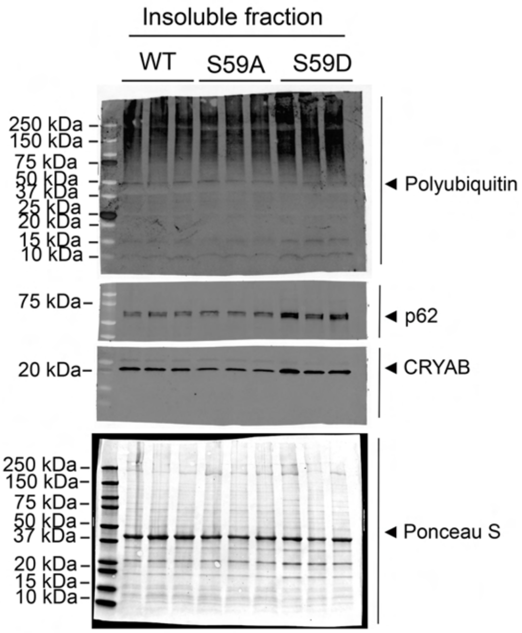

Uncropped gels western blots figure 6D

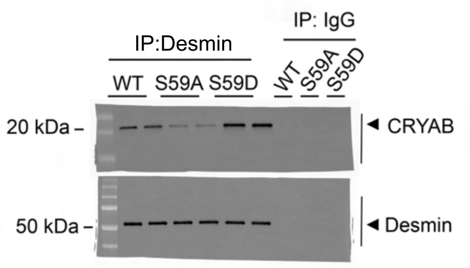

Uncropped gels western blots figure 6F

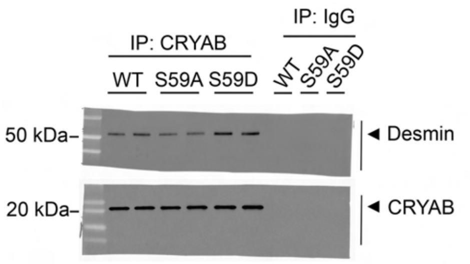

Uncropped gels western blots figure 7A

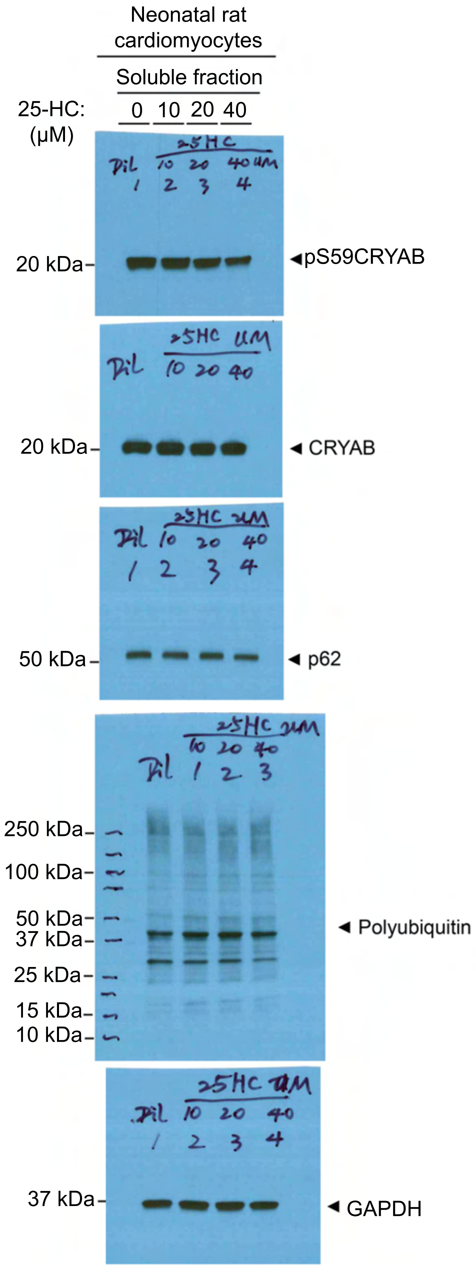

Uncropped gels western blots figure 7B

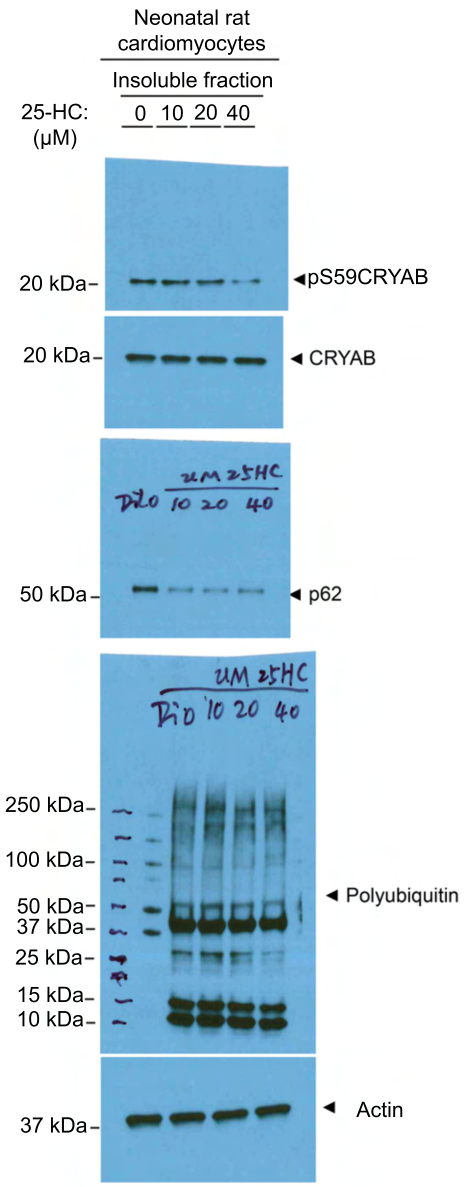

Uncropped gels western blots figure 8E

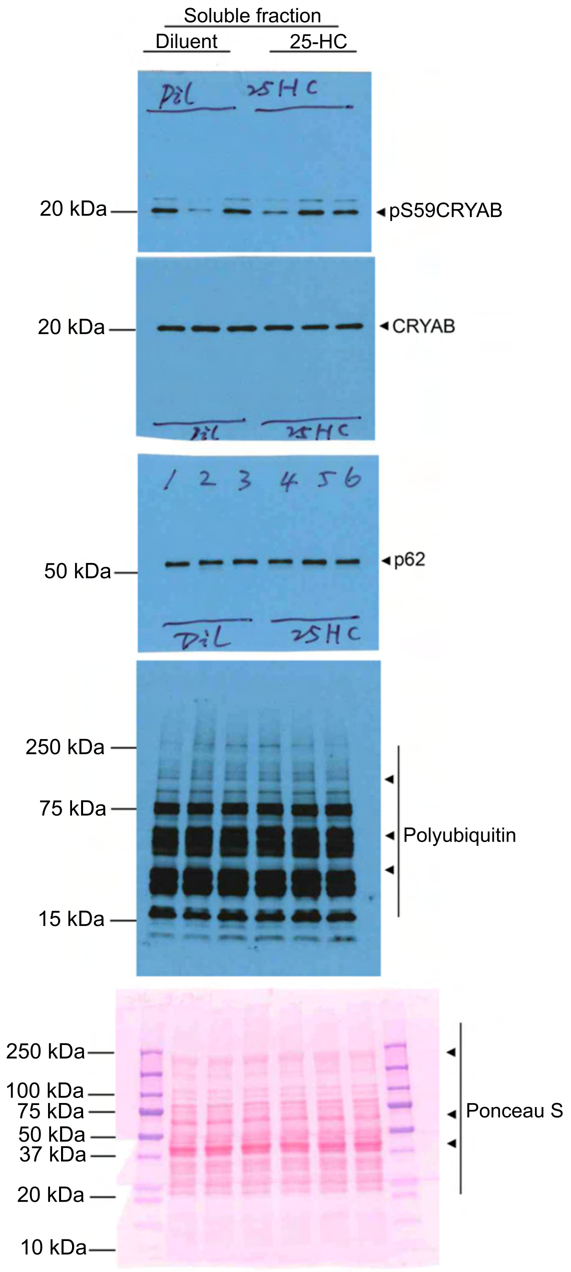

Uncropped gels western blots figure 8F

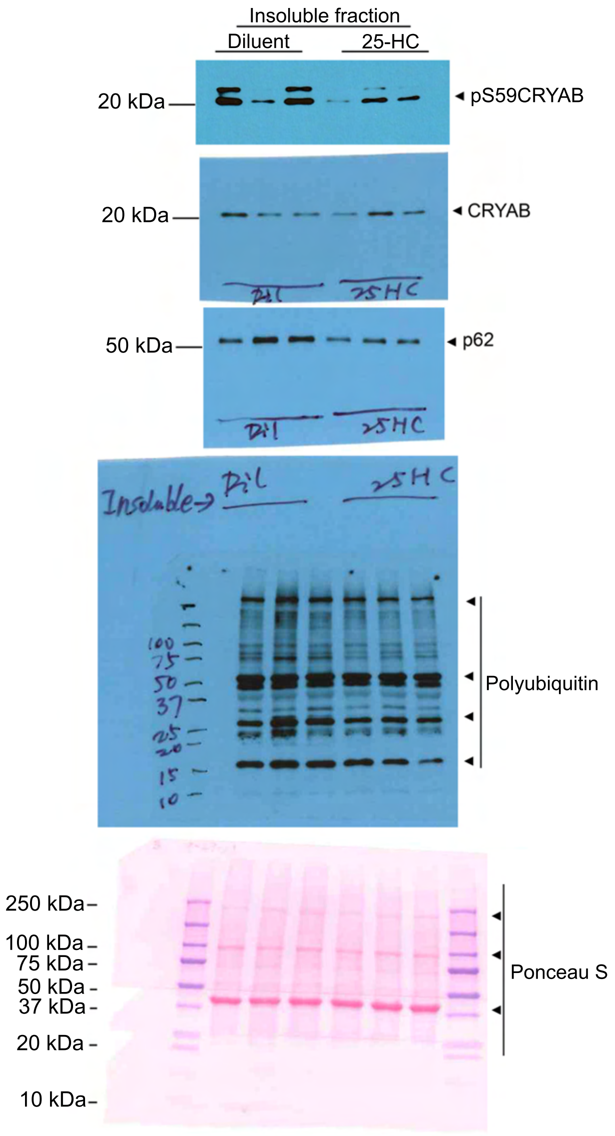

Uncropped gel western blots figure S2C

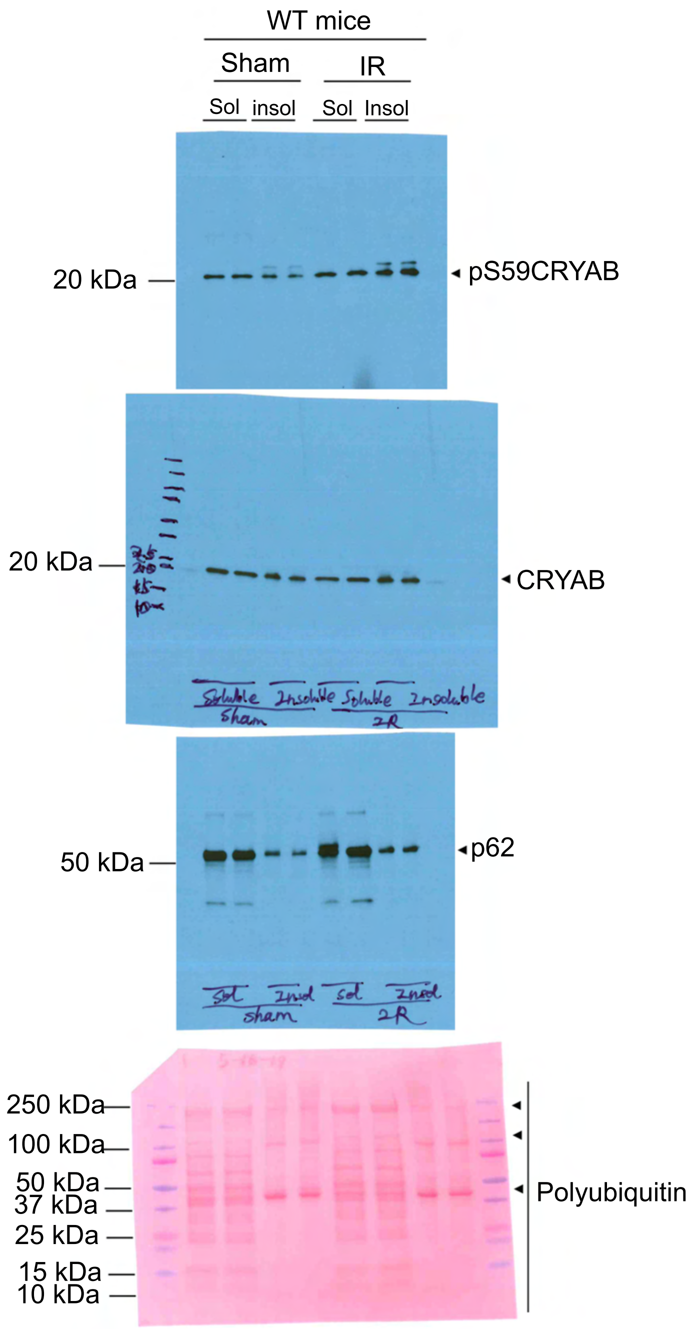

Uncropped gel western blots figure S3

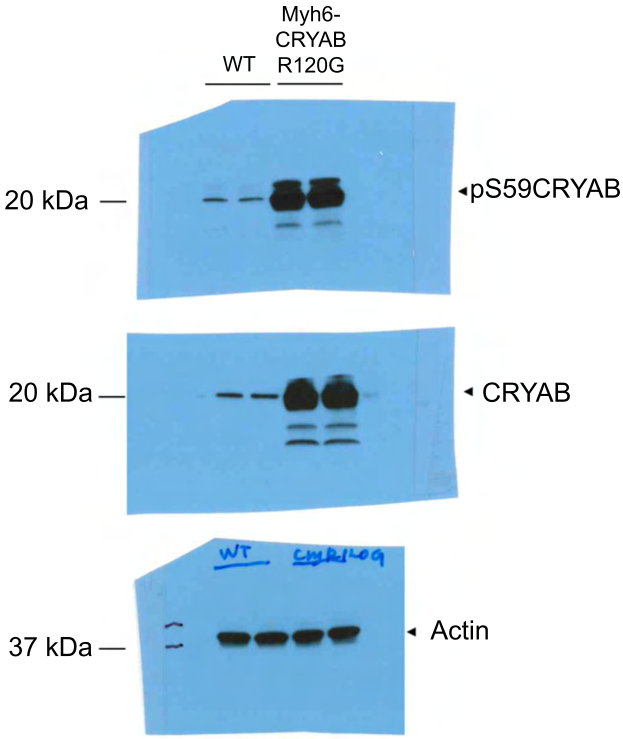

Uncropped gels western blots figure S4A

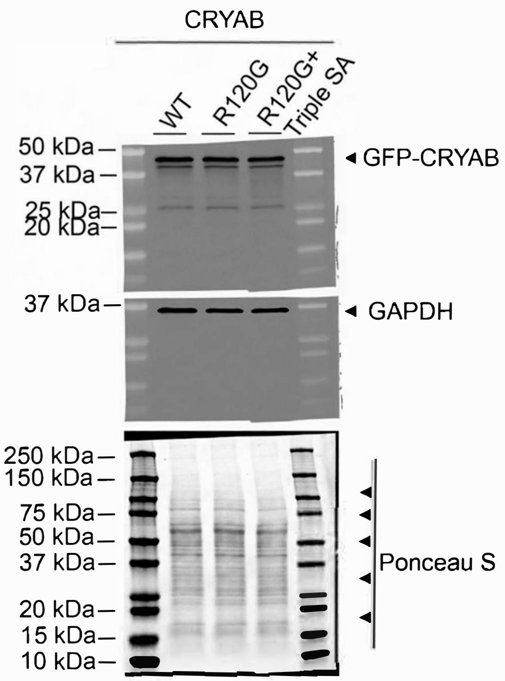

Uncropped gels western blots figure S6

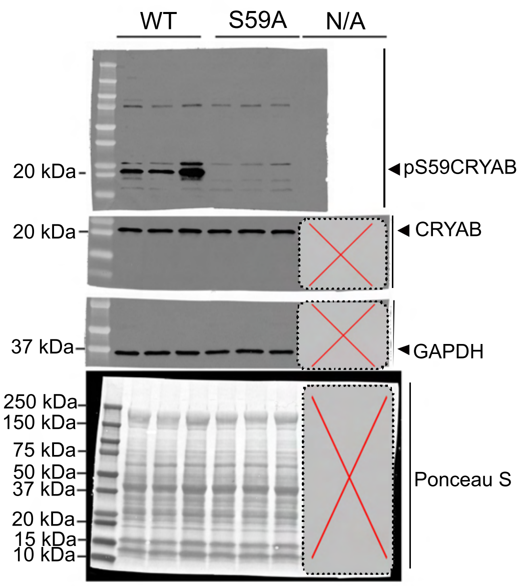

Note: N/A lanes are not applicable to this study and are marked with a cross

Uncropped gels western blots figure S7A

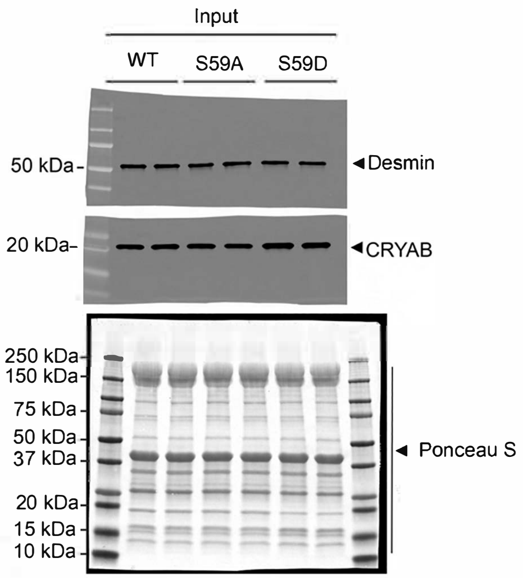

Uncropped gels western blots figure S8A

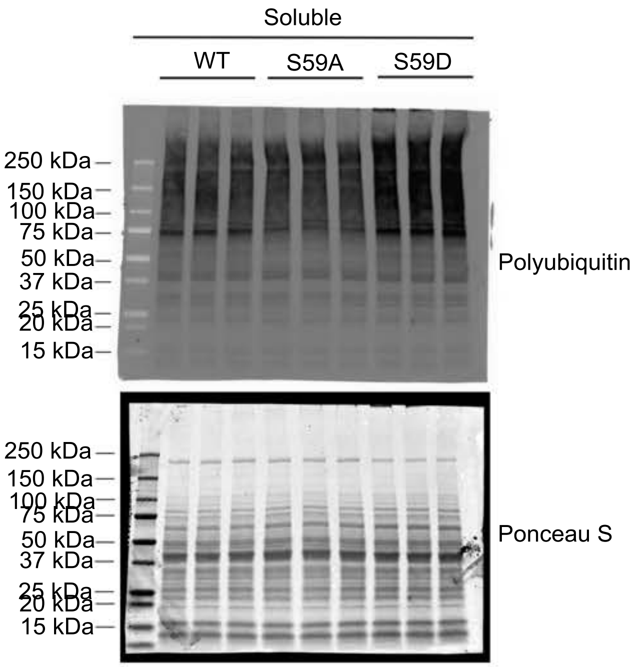

Uncropped gels western blots figure S8B

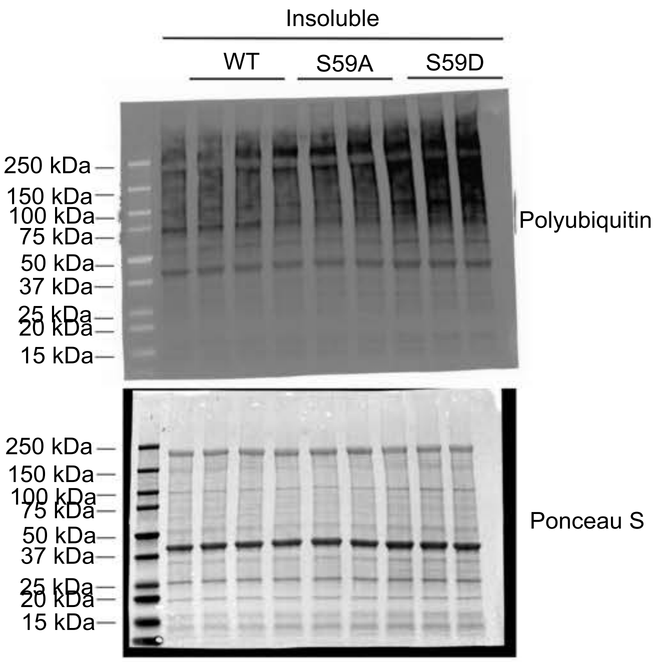

Uncropped gels western blots figure S11A

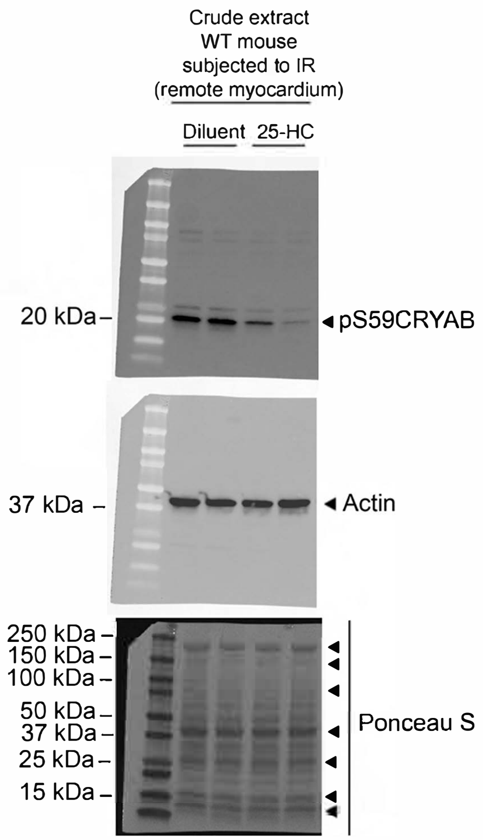

Uncropped gel western blots figure S12

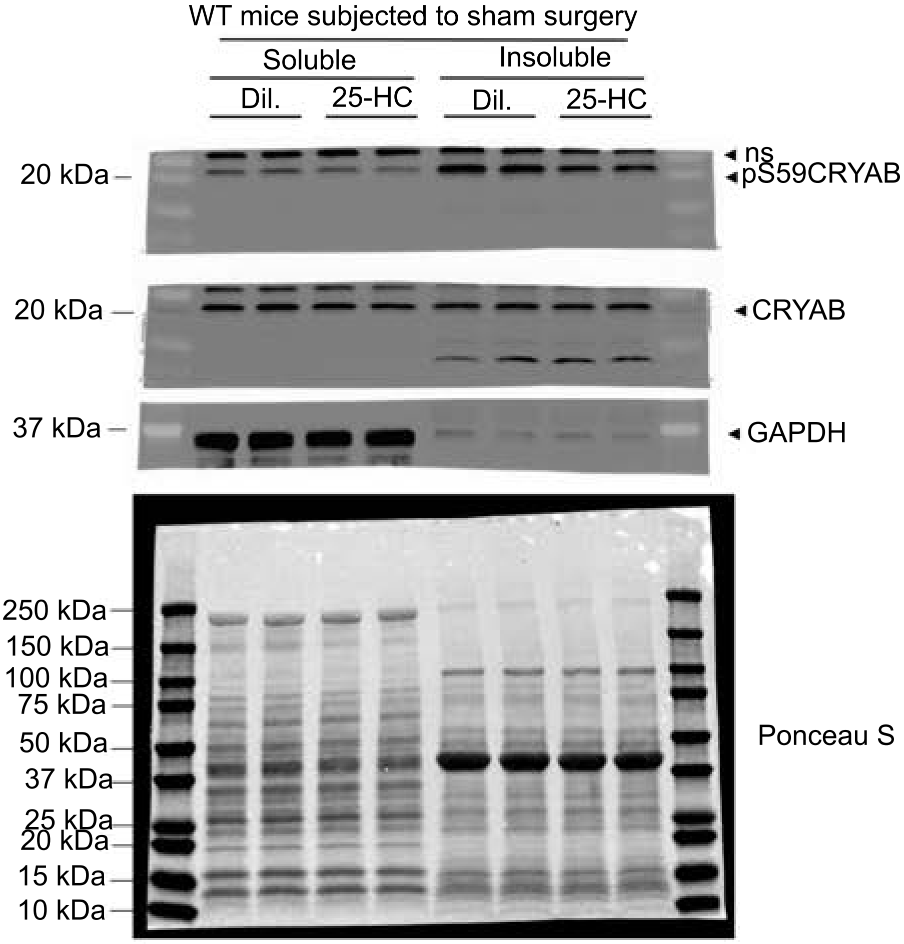

Supplement: Unedited blot and gel images [file jci-135-163730-s057.pdf]
